# EzrA promotes Z-ring formation through interaction of its QNR motif with FtsA

**DOI:** 10.1128/jb.00125-25

**Published:** 2025-07-03

**Authors:** Tingting Li, Xiujian Liu, Liangsheng Zhang, Haotian Li, Minghui Ni, Wenjin Zou, Menglei Liang, Ruotong Gong, Qiao Hu, Lelin Zhao, Zhe Hu, Lu Li, Qi Huang, Rui Zhou

**Affiliations:** 1 State Key Laboratory of Agricultural Microbiology, College of Veterinary Medicine, Huazhong Agricultural University 627716, Wuhan, China; 2 Institute of Animal Husbandry and Veterinary, Hubei Academy of Agricultural Sciences 117996, Wuhan, China; 3 International Research Center for Animal Disease (Ministry of Science & Technology of China), Wuhan, China; 4 The Cooperative Innovation Center of Sustainable Pig Production, Wuhan, China; Queen Mary University of London, London, United Kingdom

**Keywords:** cell division, *Streptococcus*, FtsZ, EzrA, FtsA, interaction

## Abstract

Bacterial cell division requires precise placement and formation of the division machinery to ensure the accurate generation of identical daughter cells. This process is canonically initiated by the highly conserved FtsZ but also needs the involvement of a variety of FtsZ-binding proteins to orchestrate the spatial and temporal positioning and assembly of the Z-ring. However, the underlying molecular mechanisms remain poorly understood. In this study, we characterized the roles of an important FtsZ binding protein EzrA in the cell division of *Streptococcus suis*, an emerging zoonotic bacterial pathogen. Our results revealed that EzrA shares high subcellular dynamics with FtsZ during the entire cell division cycle and functions primarily as a positive regulator for Z-ring formation. Co-immunoprecipitation and bacterial two-hybrid data suggest that EzrA interacts with FtsZ and several early division proteins. Importantly, the conserved QNR motif in EzrA directly contributes to its interaction with FtsA. Disrupting this motif results in the mislocalization of EzrA itself at the division site rather than the localization of FtsA, which remains concentrated localization at the division site. Moreover, the interaction of EzrA through the QNR motif with FtsA is conserved among the *Firmicutes*. Taken together, these findings demonstrate EzrA as a regulator of Z-ring positioning to the division site through the interaction of its conserved QNR motif with FtsA.

**IMPORTANCE:**

Bacteria replicate through binary fission in which the FtsZ-ring positioning and assembly is a crucial process requiring precise spatial and temporal regulation. However, the mechanism of this process remains largely unknown, especially in ovoid-shaped bacteria, such as *Streptococci,* in which many members are important human and animal pathogens. In this study, we characterize the critical role of the cell division regulator EzrA in the formation of the Z-ring. Our data reveal a model in which EzrA interacts through its QNR motif with FtsA to be properly localized to the septum so as to facilitate the positioning and formation of the Z-ring of *Streptococcus suis*. This regulatory mechanism could be conserved in *Firmicutes*. This research provides insights into the regulation mechanism of the Z-ring formation and will contribute to the understanding of the cell division process in *Streptococci*.

## INTRODUCTION

Cell division is a fundamental process that is critical for bacterial survival and proliferation, making it an important source of antibacterial targets (1). In most bacteria, cell division is accomplished by a multiprotein nanomachinery known as the divisome. Assembly of the divisome is initiated by GTP-dependent polymerization of the tubulin-like protein FtsZ at the cell midpoint to form a dynamically ring-like cytoskeletal structure, the so-called Z-ring (2–4). The dynamic Z-ring serves as a scaffold to recruit other divisome proteins to promote bacterial septation and control the sequential constitution of the cell wall biosynthesis machinery (5–8). Therefore, understanding how the Z-ring is formed and positioned is significant work that has not been fully addressed.

The FtsZ-ring is subject to elaborate regulation to guarantee its formation only at the mid-cell during the whole cell division cycle. Research on the regulation of Z-ring placement has been well studied in model rod-shaped bacteria, such as *Escherichia coli* and *Bacillus subtilis*, where Z-ring positioning occurs largely as the consequence of the coordination of the negative regulatory systems (the Min system and the nucleoid occlusion) (9, 10). Both of the systems function by inhibiting the polymerization of FtsZ outside the mid-cell region to ensure the formation of identical daughter cells (11). However, such mechanisms remain much less understood in other bacteria, especially the ovoid-shaped bacteria which do not contain these canonical negative regulation systems (12). Recent studies suggested MapZ, a *Streptococci*-specific protein, as a positive regulator for Z-ring formation in *Streptococcus pneumoniae*, which migrates toward the prospective division site coinciding with nascent treadmilling FtsZ filaments during the initial stage of division (13–15). Besides, several other FtsZ-associated proteins, including the actin homolog FtsA (16), the spectrin-like EzrA (17), the cytoplasmic proteins of ZapA, and the peripheral membrane protein SepF (18, 19), have been uncovered to coordinate Z-ring formation at the division site. However, little is known about how these regulators collaborate to effectively control Z-ring assembly.

Among these Z-ring regulators, FtsA is an important protein conserved in many species (3). The primary role of FtsA is to serve as a membrane anchor for FtsZ using its amphipathic helix at the extreme C-terminus (20). The inactivation of FtsA leads to filament cells unable to divide in *E. coli* (21), and blocks cell division with a delocalized Z-ring in *Streptococcus pneumoniae* (22). In *B. subtilis*, FtsA is only essential when GpsB or SepF is inactivated (23–25). However, in *S. pneumoniae*, FtsA is essential, and its overexpression could compensate for the abnormal cell division of GpsB or SepF deficiency (22). This suggests that the regulation of FtsA on Z-ring formation may need the involvement of other division-associated components.

EzrA is another important cell division protein related to Z-ring formation in *Streptococci* (26). It is a membrane protein comprising a single N-terminal transmembrane domain followed by a cytoplasmic topology constituted by five successive antiparallel triple-helix bundles that curve into a complete semicircle with a diameter of 120 Å (27). In *B. subtilis*, several studies revealed that EzrA functions primarily as a negative regulator for Z-ring assembly. EzrA directly interacts with FtsZ, and at least two interfaces exist between FtsZ and EzrA, one in the N-terminus of EzrA and another in its C-terminus (27–29). The primary role of the N-terminal portion of EzrA appears to function as an inhibitor for the polymerization of FtsZ filaments to higher orders *in vitro* (27, 29). The C-terminal region carries a highly conserved QNR motif responsible for the mid-cell localization of EzrA through direct interactions with membrane components or particular membrane-associated proteins, by which the mechanism remains largely unknown (28). On the other hand, research has pointed out that EzrA acts as a positive regulator for Z-ring formation in *S. pneumoniae* (30), *Staphylococcus aureus*, and *Streptococcus mutans* (31–33) since EzrA deletion in these species of *cocci* leads to enlarged spherical cells with misplaced FtsZ. Moreover, EzrA is necessary for the survival of *S. pneumoniae*, but not for *Staphylococcus aureus* and *S. mutans*. The similar velocity movement pattern of EzrA/FtsA/FtsZ filaments in *S. pneumoniae* confirms that EzrA retains intimate contact with the Z-ring (34). Moreover, apart from its role in regulating Z-ring formation, EzrA may also be involved in the tuning of the dynamic association between the nascent Z-ring assembly and peptidoglycan (PG) incorporation (35, 36).

To gain more knowledge into the precise regulation of Z-ring formation in *Streptococcus suis* compared to other *Firmicutes*, we investigate the role of EzrA in cell division and reveal that EzrA plays an indispensable role in cell morphogenesis and Z-ring position during the cell cycle in *S. suis*. Moreover, EzrA can interact directly with FtsA through its QNR motif to be located at the mid-cell and subsequently regulate the localization of FtsZ. The QNR motif-mediated interaction between EzrA and FtsA is conserved among *Firmicutes*. This study provides further insights into the involvement of EzrA in the regulation of Z-ring formation.

## RESULTS

### EzrA co-localizes with FtsZ during the cell division of *S. suis*

**Fig 1:**
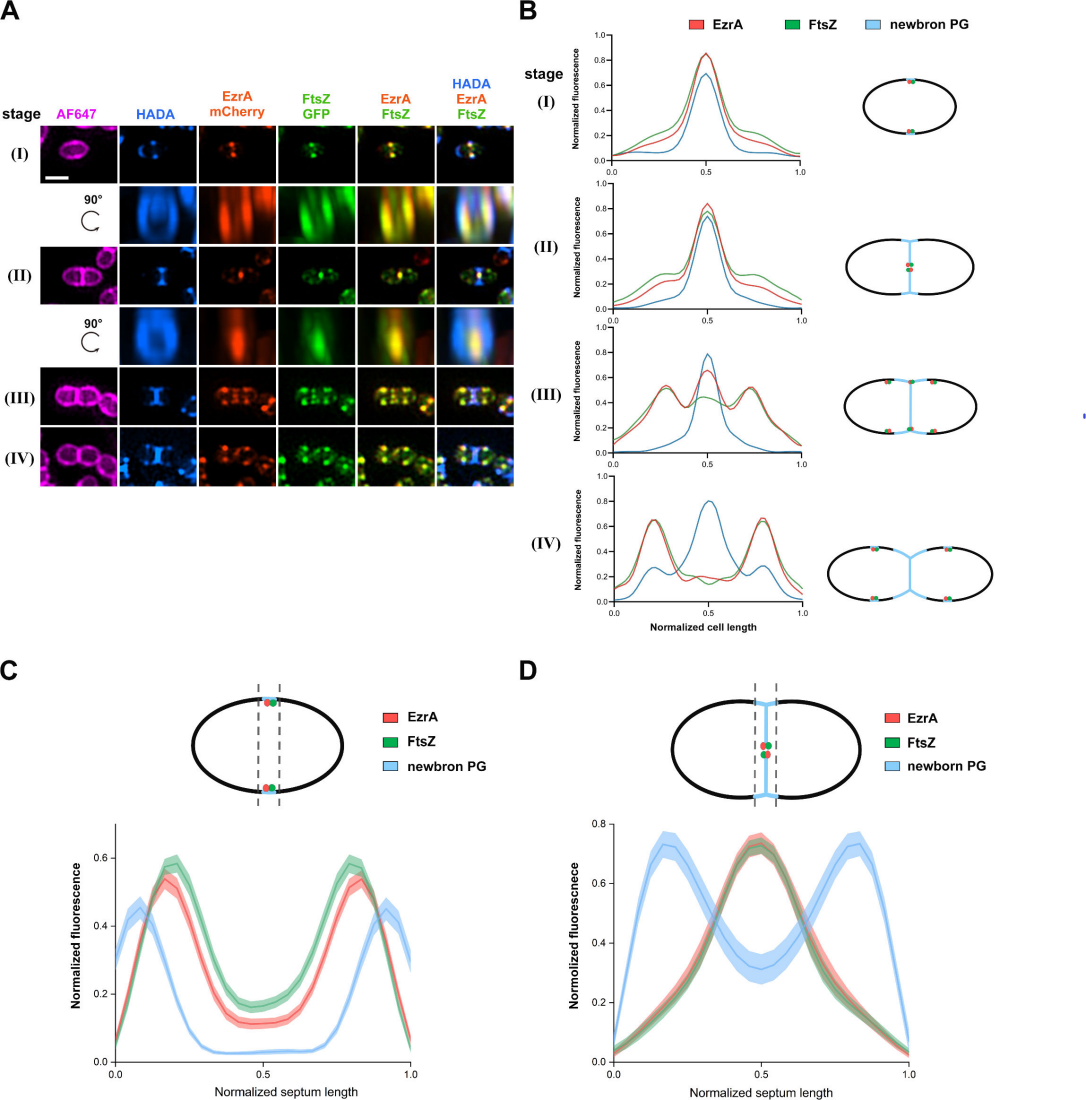
Localization of FtsZ-GFP and EzrA-mCherry in *S. suis* SC19 cells. (**A**) Representative images illustrating the subcellular localization patterns of EzrA-mCherry and FtsZ-GFP as well as nascent PG during the cell cycle. *S. sui*s SC19 cells expressing FtsZ-GFP (green) and EzrA-mCherry (red) were cultured to the mid-log phase, stained with HADA (blue) for 10 min to label nascent PG, and then stained with AF647 to indicate the cell membrane. The cells were visualized by SIM. The cells were grouped into four typical stages (I to IV) according to the morphology of the newborn PG. Rotated 3D-SIM was used to visualize the 3D patterns of the co-localization of FtsZ-GFP, EzrA-mCherry, and nascent PG for cells at stage I to stage II. Scale bar, 1 µm. (**B**) Averaged fluorescence intensity plots of HADA (blue) together with FtsZ-GFP (green) and EzrA-mCherry (red) in cells from (**A**). The fluorescence intensity was plotted against the cell length normalized from 0 to 1 (left panel). Schematics on the right panel display the localization patterns for the above three fluorescence signals along the cell length during the cell cycle. From top to bottom: *n* = 107, 51, 30, and 42 cells. (**C** and **D**) Graph of averaged fluorescence intensity of HADA (blue), FtsZ-GFP (green), and EzrA-mCherry (red) detected along lines parallel to regions confined to the septum plane in stage I cells (**C**) and stage II cells (**D**). The shaded areas suggest 95% confidence intervals. The upper panel scheme depicts the septal localization of FtsZ, EzrA, and the threshold of septal PG synthesis. Septal intensity profiles (lower panel) were normalized from 0 to 1 for each fluorescence channel plotted against the normalized distance of septum (0–1) (C, *n* = 135 cells; D, *n* = 60 cells).

To determine the subcellular localization of EzrA and its co-localization with FtsZ in *S. suis*, a fluorescent strain was constructed in which the chromosomal *ezrA* was replaced with *ezrA*-mCherry. The strain showed similar cell growth and morphology to the parental strain SC19 (Fig. S1). Then, an FtsZ-GFP fusion was produced simultaneously using an anhydrotetracycline (ATc)-inducible plasmid (37). The resulting strain was pulse-labeled with fluorescent D-alanine derivative HADA to mark newborn PG, followed by staining with Alexa Fluor 647 NHS ester (AF647) to label the cell outline and then subjected to 3D-structured illumination microscopy (3D-SIM) analysis. As displayed in Fig. 1, EzrA and FtsZ showed co-localization during the entire cell division cycle. At the start of cell division (stage I), EzrA formed a ring at the mid-cell, and the FtsZ-ring mostly overlapped with it. As the septum began concaving inward (stage II), EzrA and FtsZ showed a highly spatial correlation. At the later stage of division where the septum was closed (stage III), the occurrence of extra rings of EzrA and FtsZ was observed at the equatorial region of the future daughter cells, whereas only weak signals of both proteins could be detected at the closed septum. Finally, the majority of FtsZ and EzrA signals were seen to overlap completely at the future division site (stage IV) (Fig. 1A and B). Interestingly, the rotated 3D-SIM images also showed overlapping signals of EzrA and FtsZ (Fig. 1A). Quantitative measurement of the enrichment of three fluorescence intensity profiles along lines parallel to the septum in the cells of stages I and II indeed also suggested a high correlation between EzrA and FtsZ, confirming their co-localization (Fig. 1C and D). Whereas the width of the EzrA ring at the septum was slightly wider (but not significantly) than that of the FtsZ ring in the cells of stage I, supporting the hypothesis that EzrA might anchor FtsZ to the membrane (Fig. 1C) (26, 28). Furthermore, the diameter of the newborn septal PG ring appears to be greater laterally than that of EzrA and FtsZ rings, implying that EzrA and FtsZ may associate together to participate in the spatiotemporal regulation of septal PG synthesis (Fig. 1C and D).

### EzrA is essential for cell viability, morphogenesis, and Z-ring localization of *S. suis*

**Fig 2:**
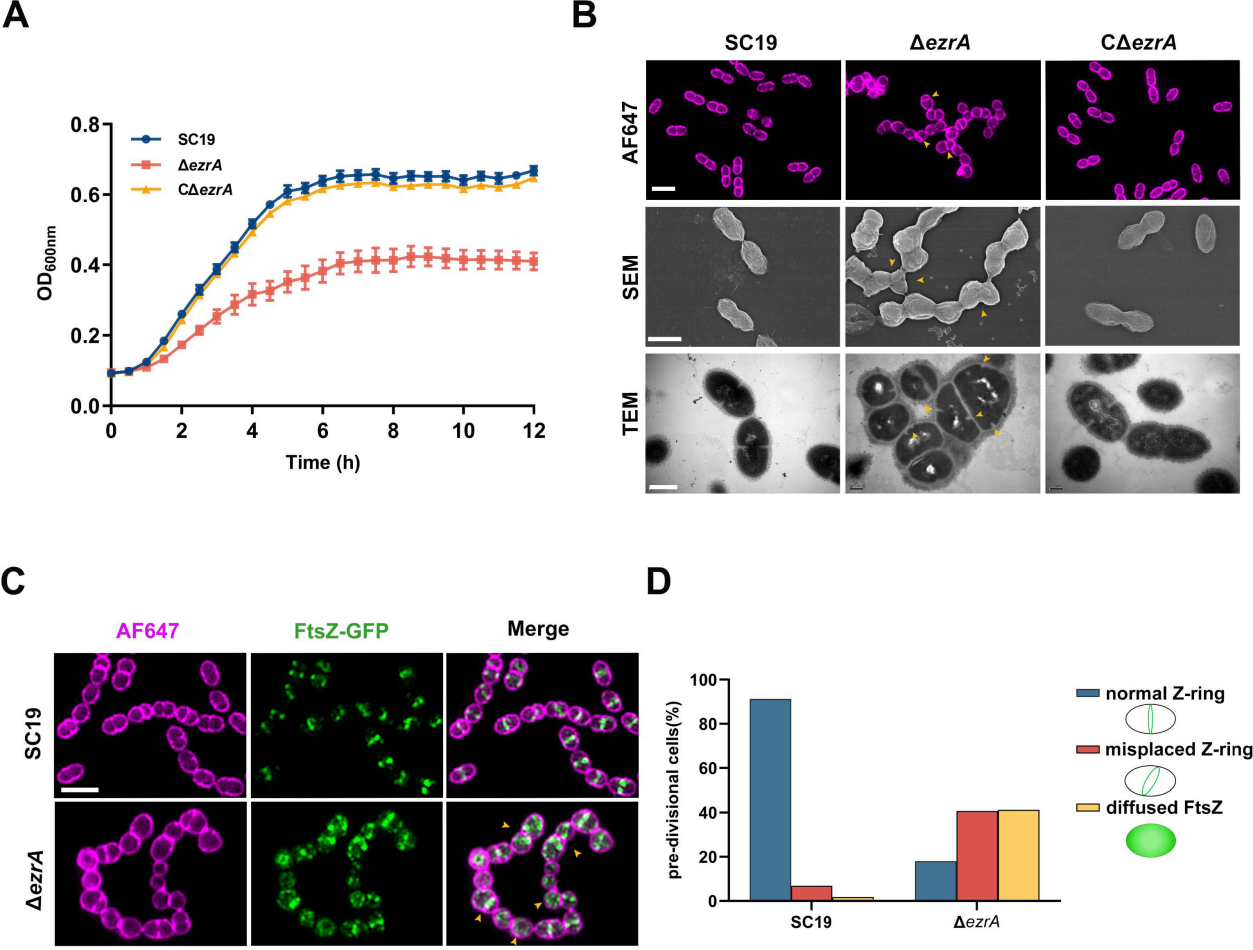
Inactivation of EzrA impairs cell viability, morphology, and Z-ring localization. (**A**) Growth curves of SC19, Δ*ezrA*, and C*ΔezrA* strain. The cells of each indicated strain were inoculated into tryptic soy broth medium from overnight grown culture and then incubated at 37°C with shaking. The OD_600_ was measured every 30 min using an automatic growth curve analyzer (**B**) Morphology analysis of SC19, Δ*ezrA,* and C*ΔezrA* cells. The cells of each indicated strain were grown to the mid-cell phase. The cells were washed and stained with AF647 dye followed by SIM imaging (upper panel, scale bar is 2 µm) and analyzed by SEM (upper panel, scale bar is 200 nm) and TEM (lower panel, scale bar is 200 nm). The yellow arrows indicate the cells with mispositioned septum. (**C**) Subcellular localization of FtsZ. The SC19 and Δ*ezrA* cells expressing FtsZ-GFP using an ATc-inducible system were grown to the mid-log phase, stained with AF647 dye, and imaged using SIM. The yellow arrows indicate the cells with mislocalized FtsZ-ring. Scale bar, 2 µm. (**D**) Statistics of normal cells, cells with misplaced Z-ring, and cells with diffused Z-ring. From left to right: *n* = 221 and 176 cells.

To directly investigate the function of EzrA in *S. suis*, a markerless *ezrA*-deletion strain (Δ*ezrA*) was generated from *S. suis* SC19. It was shown in Fig. 2A that the inactivation of EzrA resulted in a drastic decrease in growth rate, whereas the cells of the isogenic complemented strain CΔ*ezrA* showed a similar growth profile with the SC19 cells (Fig. 2A). SIM and scanning electron microscopy (SEM) analysis displayed that the Δ*ezrA* cells showed a pronounced chained and aberrant cell morphology (Fig. 2B). Transmission electron microscopy (TEM) further verified this aberrant shape of Δ*ezrA* cells which exhibited mispositioned nascent septa (Fig. 2B). Moreover, the CΔ*ezrA* strain displayed similar cell morphology to the SC19 cells (Fig. S2). Therefore, these results indicated that EzrA is not essential for survival, but indispensable for normal cell division of *S. suis*. To further evaluate the influence of EzrA on Z-ring formation, FtsZ-GFP fusion was expressed in the strains SC19 and Δ*ezrA*, respectively. SIM observation showed that in contrast to the SC19 cells in which FtsZ was normally seen in the cell midpoint, Δ*ezrA* cells showed significantly delocalized FtsZ (Fig. 2C). Only 18% of EzrA-depleted cells had a normal Z-ring at the septum, while 40.7% showed asymmetrical FtsZ localization, therefore resulting in septum misplacement. Strikingly, an accumulation of dispersed FtsZ (41.3%) around the cytoplasm and membrane was observed, indicating a role of EzrA in anchoring FtsZ to the membrane (Fig. 2D). Altogether, these results suggest that EzrA functions to spatiotemporally control the position of FtsZ during the early stage of cell division.

### Mutations on EzrA QNR motif alter septum formation and its own dynamics in *S. suis*

**Fig 3:**
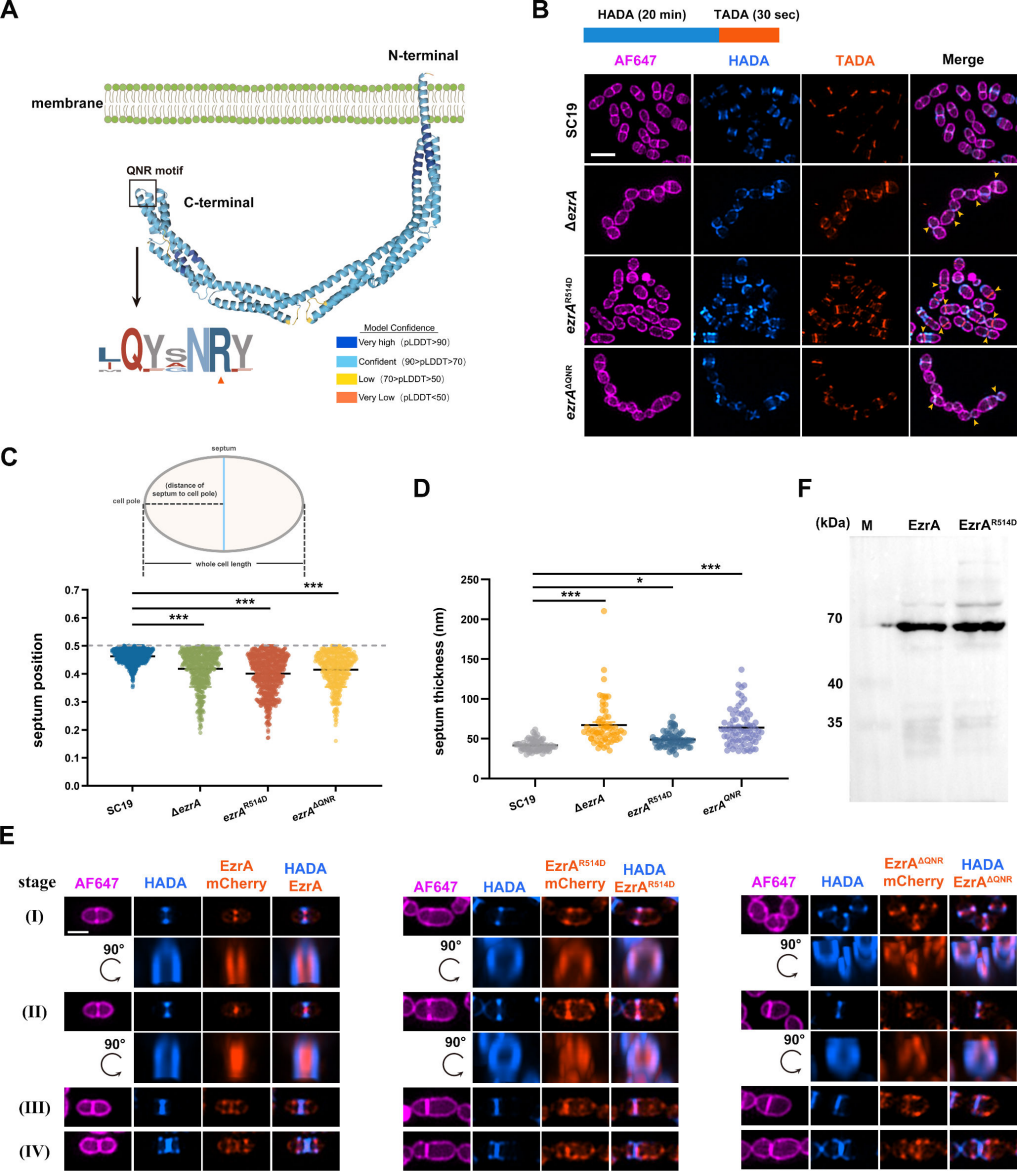
Mutations on the EzrA QNR motif impair septum and its own localization. (**A**) Structural illustration of EzrA. The full-length EzrA-3D structure was modeled using AlphaFold2 and colored by predicted Local Distance Difference Test (pLDDT) score. Sequence conservation logo of the critical QNR motif sequences was generated by WebLogo. (**B**) Morphology and PG labeling of SC19, Δ*ezrA*, *ezrA*^R514D^, and *ezrA*^ΔQNR^ cells. The cells of each indicated strain were grown to the mid-log phase. The cells were stained with a long pulse of HADA for 20 min followed by a short pulse of TADA for 30 s. The cells were then stained with AF647 to label the cell outline. The yellow arrows indicate the cells with incorrect septation. Scale bars, 2 µm. (**C**) Violin plots displaying the septum position relative to the entire cell length. Cells of *S. suis* SC19 (blue), Δ*ezrA* (green), *ezrA*^R514D^ (red), and *ezrA*^ΔQNR^ (orange) were selected from (**B**) and analyzed using ObjectJ. The relative septum position was determined by measuring the smaller distance of the cell pole perpendicular to the septation plane and divided by the whole cell length (upper panel). Gray dotted line indicated exactly mid-cell septum. From left to right: *n* = 682 (SC19), 497 (Δ*ezrA*), 673 (*ezrA*^R514D^), and 395 (*ezrA*^ΔQNR^) cells. The *P*-values were determined by one-way analysis of variance (ANOVA). ***P < 0.001. The median is indicated with a line. Each cycle indicates a single cell. (**D**) Violin plots showing the cell thickness of the septal cell wall from TEM images for *S. suis* SC19 (gray), Δ*ezrA* (orange), *ezrA*^R514D^ (blue), and *ezrA*^ΔQNR^ (purple) as analyzed using Fiji. From left to right: *n* = 71 (SC19), 64 (Δ*ezrA*), 76 (*ezrA*^R514D^), and 72 (*ezrA*^ΔQNR^) cells. The unpaired *P*-values were driven by one-way ANOVA. ***P < 0.001, *P < 0.05. The median is indicated with a line. Each cycle indicates a single cell. (**E**) Representative images displaying the localization patterns of chromosomal-expressing EzrA-mCherry (left), EzrA^R514D^-mCherry (middle), and EzrA^ΔQNR^-mCherry (right). Cells were grouped into four stages according to the nascent PG labeled with HADA. Rotated 3D-SIM images show the vertically oriented localization pattern between nascent PG incorporation and fluorescent proteins in stage I–II cells. Scale bars, 1 µm. (**F**) Western immunoblot of whole-cell lysates from SC19 and *ezrA*R514D strains as described in Material and Methods. The EzrA antibody was used to determine the presence of EzrA or its mutants.

The 3D structure of EzrA predicted by Alphafold2 showed that it shared a similar topology with its homolog in *B. subtilis*, containing a transmembrane helix in the N-terminus followed by an intracellular semi-circular structure (Fig. 3A). A highly conserved QNR motif in the C-terminus has been reported to be essential for the function of EzrA in other bacteria (26, 28). Therefore, a QNR motif-deleted strain (EzrA^ΔQNR^) and a single-mutation strain (EzrA^R514D^), in which the arginine in the QNR motif was replaced with an aspartic acid, were constructed from *S. suis* SC19. The cells were sequentially pulse-labeled with two fluorescent dyes in which HADA was stained for 20 min followed by TADA staining for 30 s to determine the dynamic PG synthesis, and followed by SIM analysis. It was shown that the majority of EzrA^ΔQNR^ cells exhibited similar morphological defects to the Δ*ezrA* cells, manifested as a severe chained phenotype with mispositioned nascent septal plane (Fig. 3B). This suggests that the QNR motif is indispensable for full function/activity of EzrA in *S. suis*. Interestingly, the asymmetrical division phenotype was more pronounced in *ezrA*^R514D^ cells in which multiple parallel septa were frequently observed in the larger daughter cells (Fig. 3B).

The relative position of the septum was determined by measuring the distance of the septum to the closer cell pole relative to whole cell length (Fig. 3C, upper panel). It was demonstrated that the SC19 cells showed a nearly mid-cell septum localization with a relative position score of 0.46, while the scores were significantly lower in the cells of Δ*ezrA*, EzrA^ΔQNR^, and EzrA^R514D^, which were 0.41, 0.41, and 0.40, respectively (Fig. 3C). To corroborate the effect of EzrA mutations on septum formation, TEM was performed to calculate the thickness of the septum (Fig. S3). Strains with mutated EzrA revealed widened septal cell walls compared to SC19 cells (41.58 nm), with an averaged thickness of Δ*ezrA,* EzrA^R514D^, and EzrA^ΔQNR^ cells of 67.17 nm, 48.92 nm, and 63.84 nm, respectively (Fig. 3D). These results suggest that the QNR motif, especially the arginine at position 514 of EzrA, is critical for correct septum formation of *S. suis*.

To investigate the role of the QNR motif in the subcellular localization of EzrA itself, two knock-in strains EzrA^R514D^-mCherry and EzrA^ΔQNR^-mCherry at its native locus were generated, which expressed the mCherry fused variant EzrA proteins in *S. suis* SC19. SIM analysis showed that both EzrA^R514D^ and EzrA^ΔQNR^ displayed aberrant spots of localization at the membrane compared to intensive localization of EzrA at the septum (Fig. 3E). Besides, rotated 3D-SIM images further demonstrated aberrant localization of both mutated EzrA proteins compared to that in SC19 (Fig. 3E). Additionally, EzrA^R514D^ was expressed in the strain *ezrA*^R514D^ at a comparable level of EzrA in SC19 (Fig. 3F). Therefore, the QNR motif of EzrA is critical for its septal localization. Taken together, the QNR motif of EzrA is critical for its localization, which may further regulate the placement of the septum.

### Mutation on EzrA QNR motif impacts dynamics of early division proteins in *S. suis*

To find clues on how the QNR motif was involved in the function of EzrA, co-immunoprecipitation (Co-IP) was then applied to compare the abundance of the interacting proteins of EzrA and those of EzrA^R514D^. Two knock-in strains of *S. suis*, EzrA-3×FLAG and EzrA^R514D^-3×FLAG, were constructed in which a triple FLAG tag was fused to the chromosomal C-terminus of EzrA or EzrA^R514D^, respectively. The interacting proteins were co-immunoprecipitated with EzrA-3×FLAG and EzrA^R514D^-3×FLAG without cross-linking, and followed by identification by mass spectrometry analysis. The results showed that the co-immunoprecipitated proteins with differential abundance were mainly those involved in the early stage of cell division (Table 1), including FtsA, ZapA, and SepF. Enrichments of SepF, ZapA, and FtsA were decreased in the EzrA^R514D^-3×FLAG strain compared to the EzrA-3×FLAG. However, there was no difference in the abundance of FtsZ, suggesting that the QNR motif may not be responsible for the interaction between FtsZ and EzrA. These data suggest a potential role for the early division proteins in recruiting EzrA to the mid-cell through its QNR motif. Surprisingly, many proteins related to septal PG synthesis showed marginally decreased enrichment in EzrA^R514D^ than EzrA (Table S1), which implies a potential association between EzrA and regulators related to septum formation (26, 38, 39).

**TABLE 1:** Cell division proteins with differential abundance co-immunoprecipitated with EzrA and EzrA^R514D*a*^

Protein	Locus	Function	Molecular mass (kDa)	EzrA^R514D^/EzrA	
				Log_2_FC^*b*^	Diff Sig^*c*^
EzrA	SSU05_1509	Z-ring formation regulator	66.43	+0.1827	NA
FtsZ	SSU05_0481	Cytoskeletal protein, core protein in cell division; principal component of the Z-ring	43.08	−0.0679	NA
FtsA	SSU05_0480	Actin-like protein, principal membrane anchor for FtsZ	50.18	−0.6	–
ZapA	SSU05_0233	Z-ring positive regulator, role in promoting Z-ring assembly	11.96	−1. 3184	–
SepF	SSU05_0484	Z-ring positive regulator, role in promoting Z-ring assembly and tethering FtsZ to membrane	21.69	−1.7313	–

^*a*^ The co-immunoprecipitation experiment was performed as described in the Materials and Methods.

^*b*^ The log_2_FC describes the differences in Label-free Quantification (LFQ) intensity immunoprecipitated from the cells expressing EzrA^R514D^ to those expressing EzrA (control).

^*c*^ Significance was determined according to the log2FC values between EzrA and EzrA^R514D^. The value (>0.263) indicates upregulated protein to form complex with EzrA^R514D^ compared to EzrA and uses a “+” to denote, while the value (−0.263) indicates downregulated protein in the complex of EzrA^R514D^ and uses a “–” to denote. “NA” indicates proteins with no detected difference.

Bacteria two-hybrid (B2H) analysis was then carried out to validate the above interaction results. A strong interaction was observed between EzrA and FtsA. Strikingly, however, a dramatically decreased interaction was seen between EzrA^R514D^ and FtsA (Fig. S4A), implying an important role of the QNR motif in EzrA for its interaction with FtsA. In contrast to the Co-IP results, there was no visible reduction in the interaction between SepF and ZapA and other division proteins (Fig. S4A). We reason that this may be attributed to the ability of EzrA in directly or indirectly recruiting these proteins to the divisome being disrupted in EzrA^R514D^-3×FLAG cells. Additionally, QNR motif mutation almost abolished the ability of EzrA to interact with other proteins, which might be related to the destabilization of its structure, as the immunoblot result showed existing bands of EzrA^ΔQNR^ fused with CyaA (Fig. S4B).

**Fig 4:**
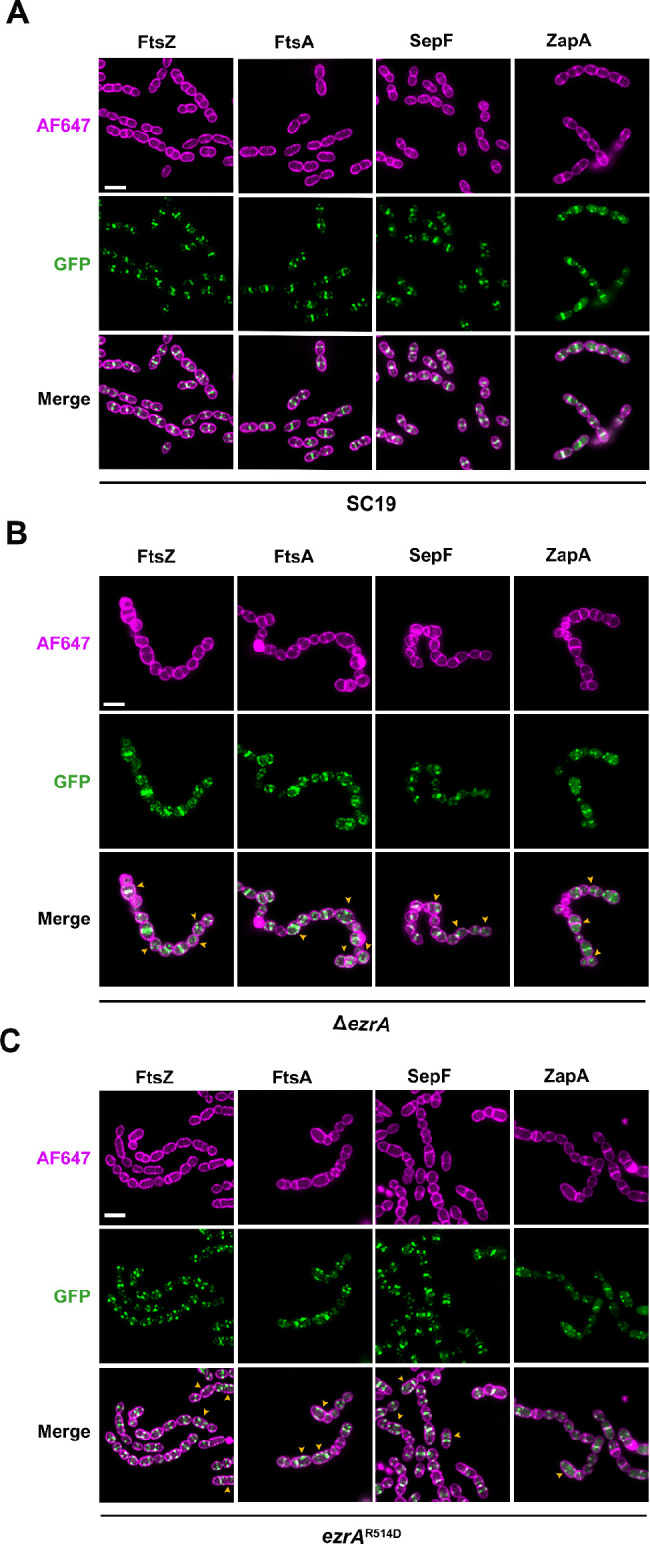
Subcellular localization of FtsZ, FtsA, SepF, and ZapA in SC19, Δ*ezrA*, or *ezrA*^R514D^ cells. GFP-fused FtsZ, FtsA, SepF, or ZapA was expressed in SC19 (**A**), Δ*ezrA* (**B**), or *ezrA*^R514D^ (**C**) cells, respectively, using the ATc-inducible expression system. The cells were stained with AF647 dye to label the cell outline and then analyzed by SIM. The yellow arrows indicate the cells with aberrant localization of GFP-fused proteins. Scale bar, 2 µm.

Since mutation in the QNR motif of EzrA decreased its interaction with the early division components, including SepF, ZapA, and FtsA, we next investigated the influence of mutation in the QNR motif on the subcellular localization of these proteins. Plasmid-encoded GFP fusion of FtsZ, FtsA, SepF, and ZapA was expressed in SC19, Δ*ezrA*, and EzrA^R514D^ strains, respectively, and their subcellular localizations were determined by SIM analysis. All four GFP-fused proteins exhibit exclusive locations at the division septum in the SC19 cells (Fig. 4A). In EzrA-deleted cells, FtsZ, FtsA, SepF, and ZapA all showed frequent delocalization from septum to the membrane (Fig. 4B). Moreover, in EzrA^R514D^ cells, although those proteins maintain concentrated foci at the closing septum, multiple incomplete rings of them at the larger future daughter cells caused by asymmetry division can be frequently seen (Fig. 4C). These localization defects of the early division proteins caused by either the absence of EzrA or septal mislocalization due to the EzrA^R514D^ variant confirm that EzrA contributes to coordinate the localization of those early division proteins during the cell cycle.

### FtsA recruits EzrA to the septum through the QNR motif during *S. suis* cell division

**Fig 5:**
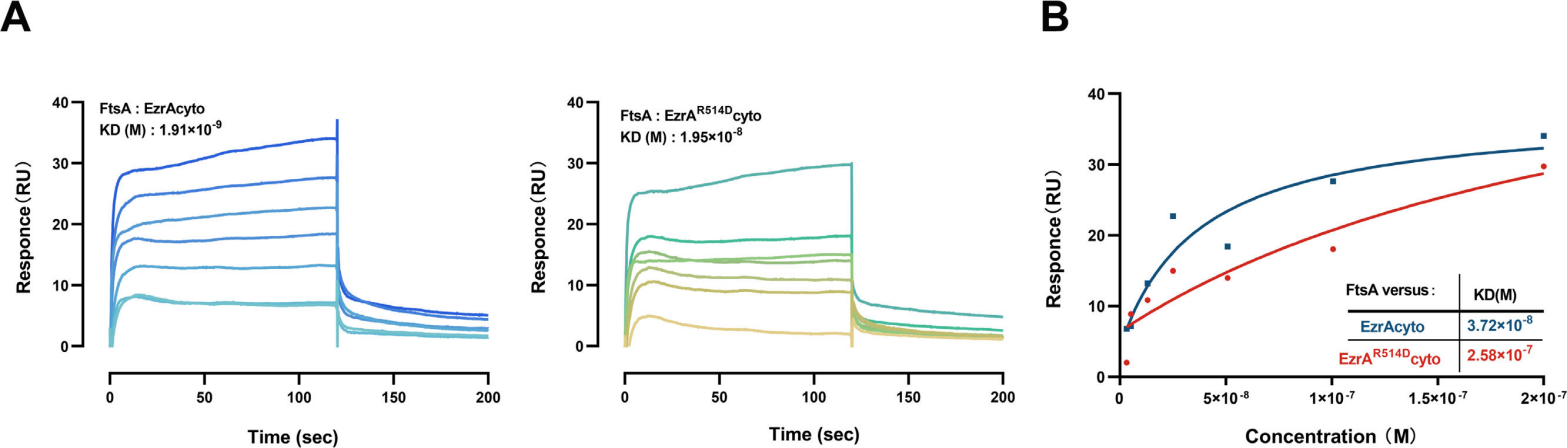
SPR analysis of the interaction between FtsA and EzrA proteins *in vitro*. (**A**) Quantification of binding of EzrA (left panel) or EzrA^R514D^ (right panel) to immobilized FtsA was determined by SPR. Gradient concentrations of analyte proteins were flowed over a CM5 chip with the immobilized FtsA. The concentration range for EzrA or EzrA^R514D^ was used from 200 µM (dark blue for EzrA or dark green for EzrA^R514D^) to 3.125 µM (light blue for EzrA or light yellow for EzrA^R514D^). RU, resonance units. (**B**) The response units of EzrA (blue) or EzrA^R514D^ (red) binding to immobilized FtsA were fitted to the equilibrium resonance signal corresponding to a 1:1 binding mode.

The above results have revealed that EzrA interacts with FtsA, and QNR motif mutation significantly disrupted this interaction. The binding affinity between EzrA and FtsA was further characterized *in vitro* by using surface plasmon resonance (SPR) assay (Fig. S5). It was shown that both EzrA and EzrA^R514D^ can bind to FtsA with rapid kinetics (Fig. 5A), allowing measurement for equilibrium binding level. However, the fitted binding isotherm to a 1:1 binding model showed that EzrA displayed at least 10-fold faster binding affinity to FtsA compared to EzrA^R514D^ (Fig. 5B). Such binding discrepancy supports the need for the QNR motif of EzrA to mediate its binding affinity to FtsA.

**Fig 6:**
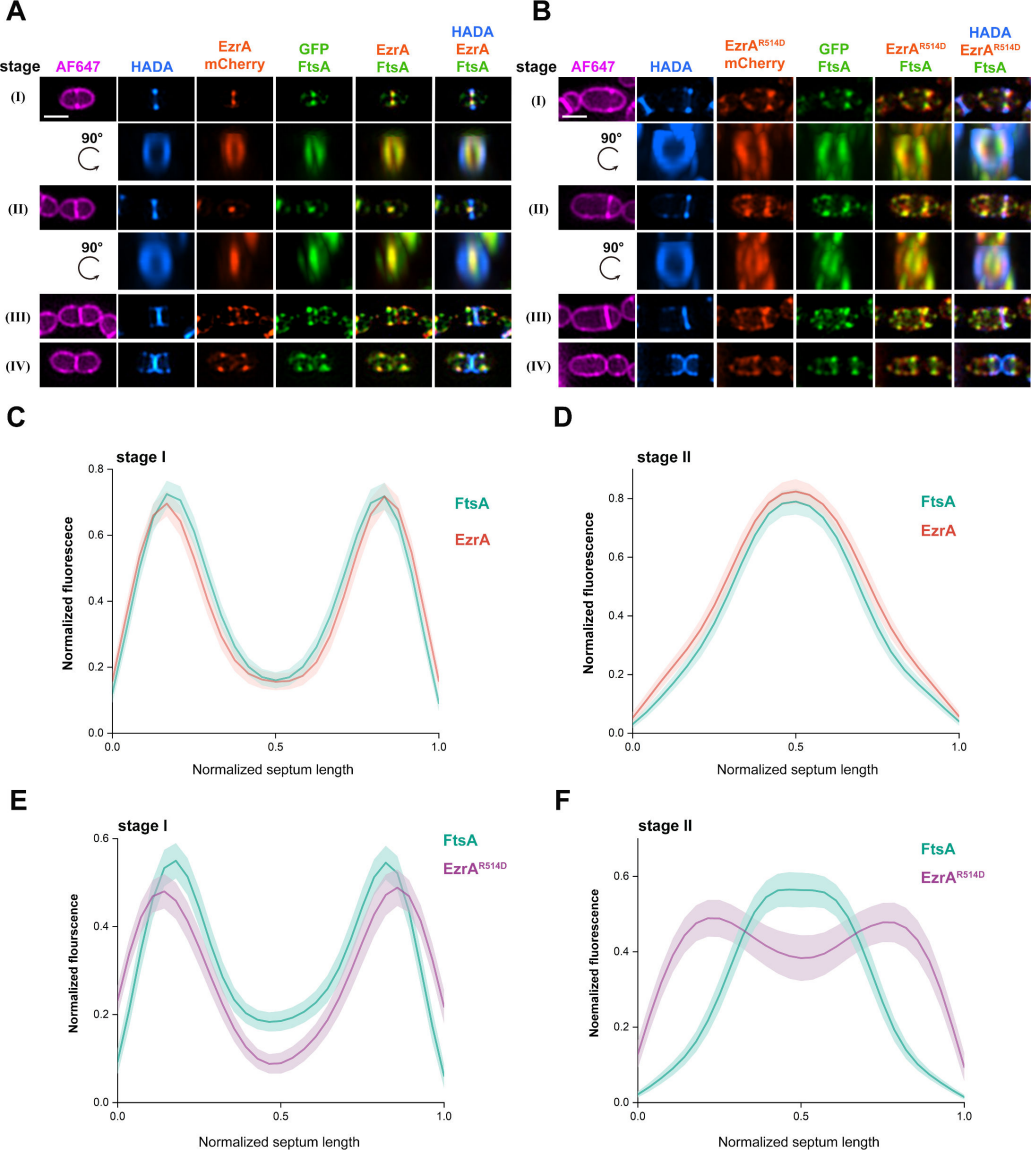
Subcellular localizations of EzrA and FtsA are damaged in EzrA^R514D^ cells. Co-localization patterns of EzrA (**A**) or EzrA^R514D^ (**B**) with FtsA. Strains with chromosomal expression of EzrA-mCherry (**A**) or EzrA^R514D^-mCherry with ATc-inducible expression of FtsA-GFP were grown to the mid-log phase. The cells were stained with HADA for 10 min, followed by staining with AF647. The cells were imaged by SIM and were grouped into four stages according to the morphology of the nascent PG. Rotated 3D-SIM images display the vertically oriented localization modes of GFP-FtsA and EzrA-mCherry or EzrA^R514D^-mCherry, along with pulsed-labeled HADA at the septal panel in stage I cells and stage II cells. Scale bar, 1 µm. (**C and D**) Graphs of mean fluorescence intensity of GFP-FtsA (green) and EzrA-mCherry (red) collected at the septum region in stage I cells (**C**) and stage II cells (**D**). Shaded areas show 95% confidence intervals. (**E and F**) Graphs of fluorescent intensity profiles of GFP-FtsA (green) and EzrA^R514D^-mCherry (purple) collected at the septum region in stage I cells (**E**) and stage II cells (**F**) were analyzed. The mean intensity profiles (C, D, E, and F) were normalized from 0 to 1 for each fluorescence channel plotted against the normalized septum length (0–1) (C, *n* = 110 cells; D, *n* = 77 cells; E, *n* = 106 cells; F, *n* = 74 cells).

Since mutation in the QNR motif decreased the binding of EzrA to FtsA in *S. suis*, co-localization analysis was further performed between FtsA and EzrA or EzrA^R514D^, respectively. Plasmid-encoded GFP-FtsA fusion was introduced into the strain expressing EzrA-mCherry and EzrA^R514D^-mCherry, respectively, and then analyzed by microscopy. It was seen that the fluorescent signals of GFP-FtsA and EzrA-mCherry showed complete overlap during the cell cycle (Fig. 6A), indicating their highly temporal and spatial association during cytokinesis. However, rotated 3D-SIM images revealed that the EzrA^R514D^-mCherry did not show a co-localized pattern with GFP-FtsA at the septal plane (Fig. 6B). Other than the exclusively concentrated foci of GFP-FtsA at the inner edge of the newborn septal PG, the fluorescent signal of EzrA^R514D^-mCherry not only occurred at the inner face of the nascent septal PG ring, but also appeared to overlap with it (Fig. 6B). To further strengthen the above observation, we quantified fluorescent enrichment along lines paralleled to the septal plane in the cells of stages I and II. The fluorescence of EzrA and FtsA displays the same enrichment during the septation of SC19 cells (Fig. 6C and D). However, this co-enrichment pattern between EzrA and FtsA stands in stark contrast to the significantly detached enrichments for EzrA^R514D^-mCherry and GFP-FtsA at the septal plane, where EzrA^R514D^ seems to be mainly enriched at the outer edge of FtsA (Fig. 6E and F). These data reinforce the model that FtsA governs the mid-cell localization of EzrA.

### The essentiality of the QNR motif on Z-ring formation is conserved among *Firmicutes*

**Fig 7:**
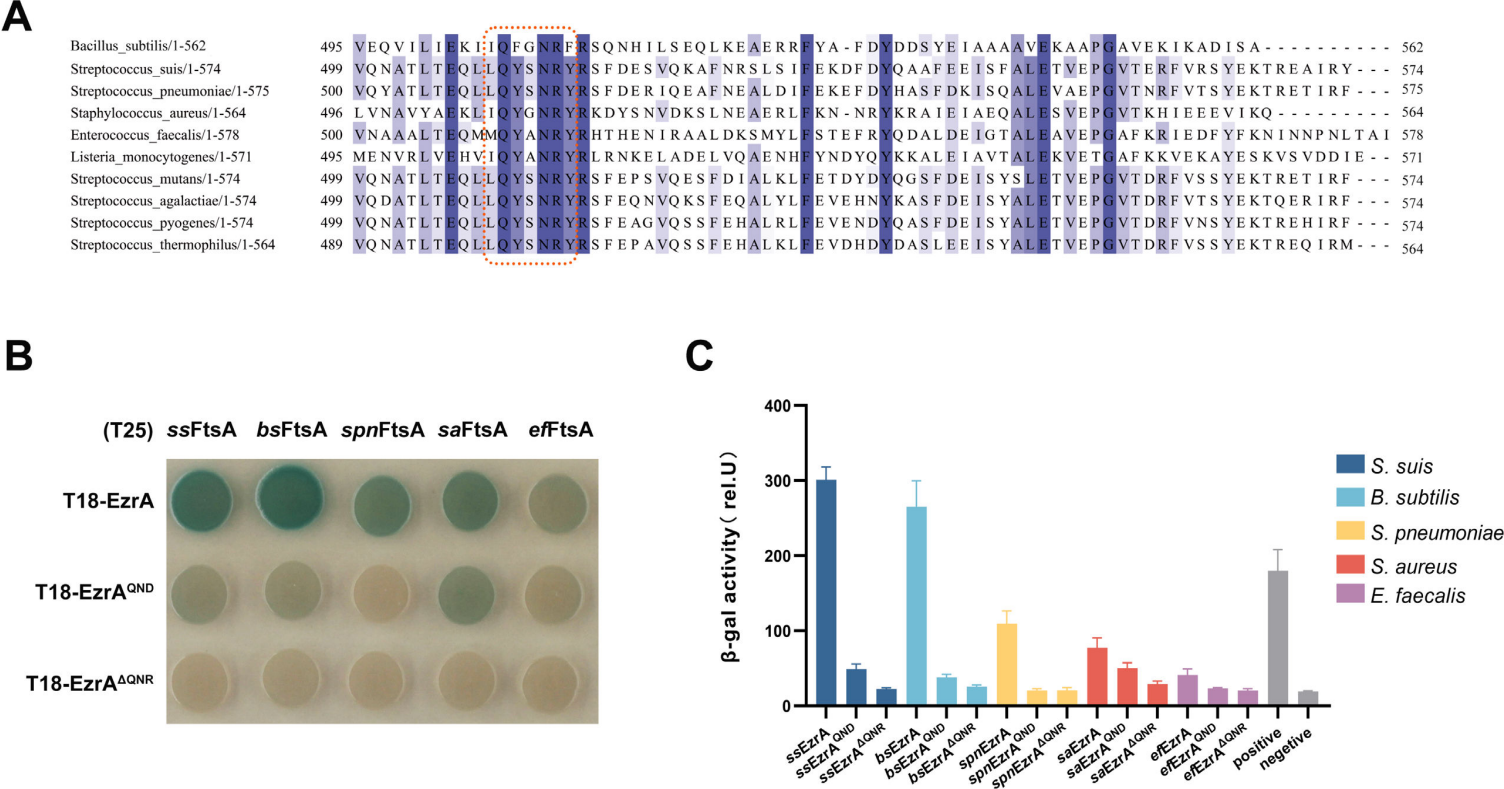
Validation of the interaction between homologs of FtsA and EzrA among *Firmicutes*. (**A**) Multiple sequence alignment of EzrA C-terminal sequences. The C-terminal sequences of EzrA from the indicated strains were extracted, which were subjected to analysis using Clustal Omega and presented using Jalview with the conserved QNR motif highlighted by a red-dashed box. (**B**) Bacterial two-hybrid assay. *E. coli* BTH101 cells expressing each indicated T18- and T25-fused proteins were spotted onto Luria-Bertani (LB) agar plates containing chloramphenicol, ampicillin, streptomycin, X-Gal, and isopropyl β-D-thiogalactoside (IPTG), followed by incubation at 30°C in the dark for 24 h. *ss*FtsA, FtsA of *S. suis; bs*FtsA, FtsA of *B. subtilis; spn*FtsA, FtsA of *S. pneumoniae; sa*FtsA, FtsA of *S. aureus; ef*FtsA, FtsA of *E. faecalis*. Same as the FtsA versions in the T25 constructs, the EzrA versions in the T18 constructs are from different species, corresponding to that of the FtsA. (**C**) Quantitative analysis of the interactions for B2H. Cells containing pUT18- and pKT25-derivative plasmids were cultured at 30°C for 16 h. Then the β-galactosidase activity was measured as described in the Materials and Methods. The interaction pair of pUT18-zip and pKT25-zip was used as a positive control, while the pUT18 and pKT25 pair was used as a negative control.

Protein alignment analysis revealed that EzrA is a conserved protein in *Firmicutes,* and its QNR motif is also conserved (Fig. 7A). Thus, we examined whether the QNR motif of EzrA played an important role in mediating interaction with FtsA as well as Z-ring formation in other species of *Firmicutes*. Firstly, the EzrA-FtsA interaction was characterized by using the B2H assay. It was shown that this interaction was also detected in *B. subtilis*, *S. aureus*, *S. pneumoniae,* and *Enterococcus faecalis*, and lack of QNR motifs disrupted such interaction (Fig. 7B). Then, quantitative analysis by β-galactosidase activity further corroborated the interaction. Notably, deletion of the QNR motif or mutation of the arginine (R) residue in *S. pneumoniae* almost abolished the interaction between EzrA and FtsA (Fig. 7C).

**Fig 8:**
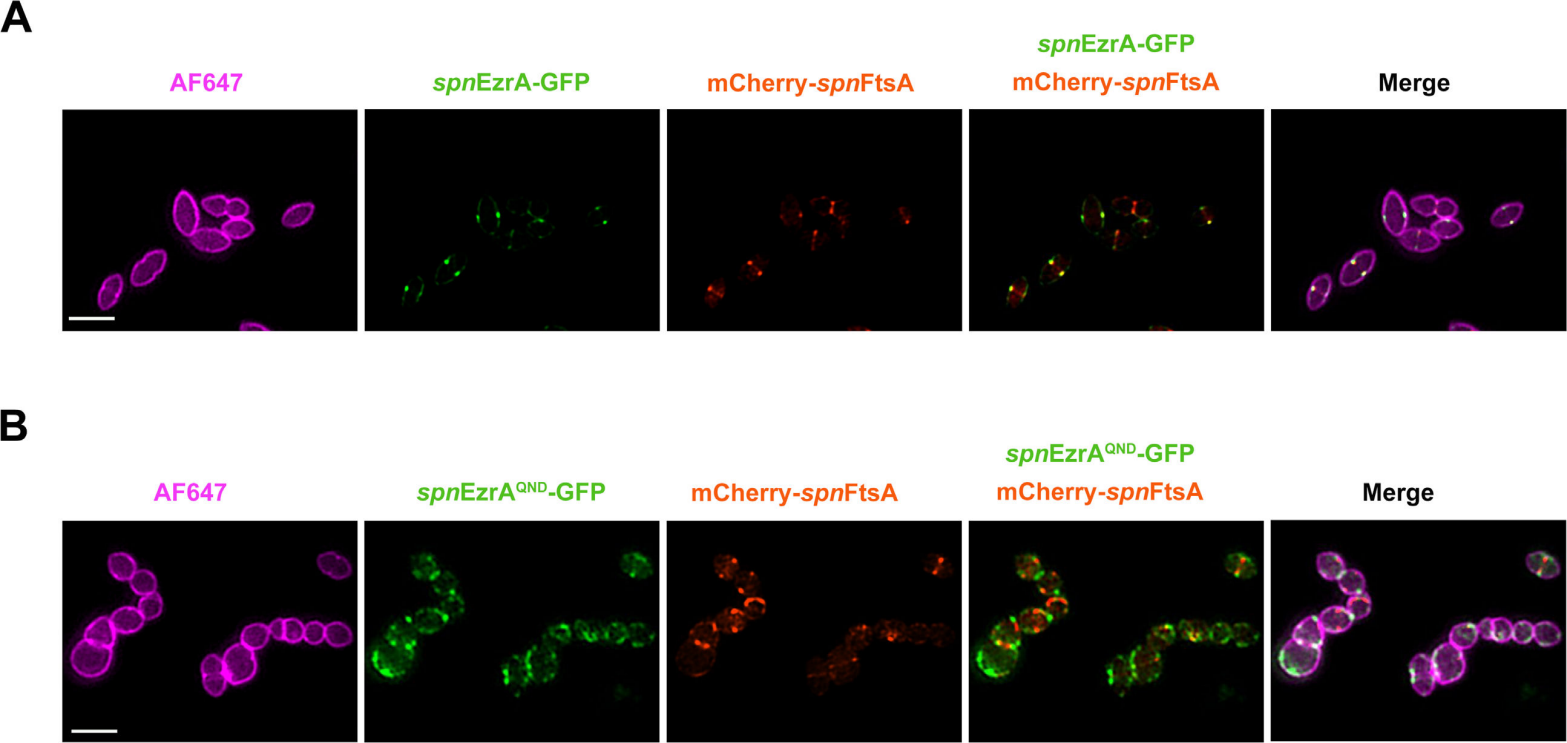
*spn*EzrA and *spn*FtsA subcellular localizations are damaged in *spn*EzrA^QND^ cells. Co-localization of mCherry-*spn*FtsA with either *spn*EzrA-GFP (**A**) or *spn*EzrA^QND^ in *spn*EzrA^QND^ cells (**B**). *S. pneumoniae* D39Δ*cps rpsL*^+^ with chromosomal expression of mCherry-FtsA (Zn^2+^-inducible) together with EzrA-GFP or EzrA^QND^-GFP was grown to the mid-log phase and stained with AF647 followed by SIM analysis. Scale bar, 2 µm.

At last, the co-localization pattern of FtsA with EzrA or EzrA^QND^ was explored in *S. pneumoniae*. To this end, an *S. pneumoniae* strain was constructed by replacing the chromosomal *ftsA* with a mCherry-*ftsA* fusion. However, this strain showed aberrant cell growth and morphology, indicating that the chromosomal mCherry-*ftsA* fusion was not fully functional (Fig. S6A and B). Then, an mCherry-*ftsA* fusion under the control of the Zn^2+^-induced promoter was integrated at the non-essential *bga* locus of *S. pneumoniae*. SIM analysis showed that the mCherry-*spn*FtsA was properly localized at the mid-cell (Fig. S6C and D). Then, *gfp* was fused to the C-terminus of the chromosomal *ezrA,* which showed normal expression and no detrimental effect on the cell growth or morphology (Fig. S7). Therefore, the pneumococcal strains with Zn^2+^-inducible expression of mCherry-*spn*FtsA together with *spn*EzrA-GFP or *spn*EzrA^R515D^-GFP were constructed, respectively. It was observed that *spn*EzrA and *spn*FtsA showed completely overlapped fluorescent signals at the division site similar to that of *S. suis* (Fig. 8A), indicating their intimate co-localization during the cell cycle. Importantly, it was revealed that the dynamic of *spn*EzrA^QND^-GFP was affected as it was distributed diffusely throughout the cell membrane (26). However, mCherry-*spn*FtsA remains exclusively localized at the division site (Fig. 8B). These results indicate that the QNR motif in EzrA is the key determinant for its septal localization but does not interfere with the proper localization of FtsA in *S. pneumoniae*.

## DISCUSSION

Bacterial cell division requires precise assembly and placement of the Z-ring to guide septal development for partitioning. The correct formation and localization of the Z-ring relies on the elaborate coordination of multiple FtsZ-associated proteins. Several FtsZ-associated proteins are conserved in gram-positive bacteria, including FtsA, EzrA, ZapA, and SepF (40). Notably, in *B. subtilis*, none of the single deletion mutants is lethal. However, synthetic lethality and failure of Z-ring condensation were observed when EzrA was simultaneously deleted with SepF or ZapA (31), indicating critical interplay among these proteins in coordinating proper Z-ring formation. Therefore, understanding how these FtsZ-associated proteins work with each other to ensure the accurate placement of the Z-ring is critical for understanding the cell division mechanism.

In this study, we first investigated the association between EzrA and FtsZ in *S. suis* and uncovered that these two proteins spatiotemporally function together during the cell cycle. An obvious morphological feature of *ezrA*-deleted cells is the irregular shape accompanied by mispositioned septum. Notably, in *S. suis*, the *ezrA*-deleted mutant exhibits an abnormal Z-ring, manifesting as a single skewed Z-ring or dispersed FtsZ among cytoplasm or cell membrane, which is in contrast to the delocalized FtsZ with the extra Z-rings at the cell pole in *B. subtilis* (33). This underscores a positive role of EzrA in the Z-ring position at the mid-cell resembled that in *S. aureus* (32), *S. pneumoniae* (26), and *S. mutans* cells (41). However, EzrA was considered to possess separably contradictory effects on the Z-ring in *B. subtilis* (31). One effect is to inhibit FtsZ polymerization *in vitro* and shorten FtsZ filaments *in vivo*, and the other is in conjunction with SepF and ZapA to positively regulate Z-ring formation at the division site. Actually, in this work, the observed slight distribution of EzrA at the membrane, combined with increased FtsZ localization dispersed around the cytoplasm, may be in agreement with the negative role of EzrA in binding free FtsZ monomers to restrain Z-ring formation at the non-division site, as EzrA deletion induces dispersed localization of FtsZ around the cytoplasm (Fig. S1C and 2D) (26). On the other hand, the mid-cell localized EzrA may collaborate with other regulators to positively stabilize Z-ring placement.

The mechanism of EzrA itself to localize at the division site is a critical prerequisite for deciphering how EzrA acts to coordinate the Z-ring position at the cell midpoint. Previous studies have pointed out the crucial role of the conserved QNR motif of EzrA for its function, by which this motif may mediate the interaction of EzrA with some important but unidentified proteins to govern its mid-cell localization (26, 28). Consistently, our results revealed that mutation on the QNR motif indeed altered the subcellular localization pattern of EzrA itself in *S. suis* (Fig. 3E). Moreover, the mutated strain of EzrA^R514D^ also displayed a mispositioned Z-ring due to misplaced EzrA at the septum. These results stress the notion that the QNR motif is dispensable for EzrA’s full function (26). We further revealed that FtsA acts as a cell midpoint anchor for EzrA through direct interaction with the QNR motif of EzrA, in which the arginine influences their binding affinity. This regulatory mechanism is reported for the first time in species containing EzrA. As one of the earliest proteins to arrive at the division site, FtsA serves as the primary Z-ring membrane tether (20). Depletion of FtsA leads to dispersed localization of FtsZ (22, 42), an observation partially similar to EzrA-deleted cells of *S. suis* and *S. pneumoniae* (26). This finding, thus, fits well with an auxiliary role for EzrA to tether FtsZ to the membrane. On the other hand, in *S. pneumoniae*, the nascent bundles of FtsZ/EzrA/FtsA migrate to the future equators with the same velocity, further confirming their indiscerptible contact to guarantee normal cell division (34). This intimate association is best elucidated by the co-localization pattern of EzrA and FtsA during cytokinesis in *S. suis*, as well as the observation that mutation in the key arginine residue of EzrA’s QNR motif can alter its co-localization with FtsA. In contrast, the dynamics of FtsA at the septum are not changed despite the QNR motif mutation of EzrA, implying that FtsA is responsible for recruiting EzrA to the division site to collectively stabilize the Z-ring formation.

Another important finding of this work is that the regulatory mechanism by which FtsA controls the midpoint position of EzrA is broadly conserved among *Firmicutes*. This conserved mechanism can be rationalized by considering at least two perspectives based on current research. First, EzrA and FtsA evolutionarily maintain conserved topology (16, 27), even though these species differ largely in morphogenesis and Z-ring regulation systems involved in Z-ring placement. Second, aside from collectively stabilizing the Z-ring position, actually, EzrA and FtsA also have overlapping functions in contacting other cell division proteins to orchestrate the divisome and promote the progression of PG synthesis in various bacteria (26, 43). For instance, in *S. pneumoniae*, FtsA functionally links with GpsB to coordinate septal and peripheral PG synthesis (22). Similarly, it has been demonstrated that EzrA acts as an intermediate coordinator of septum formation via forming a dynamic complex with FtsZ and GpsB (35, 39). Moreover, our finding suggests that the thick septal cell wall in cells with delocalized EzrA at the division site may be attributed to reduced recruitment of SepF, as SepF was previously shown to control septal thickness in gram-positive bacteria (44, 45). These conserved cross-overlapping functions between EzrA and FtsA further underline their intimate interplay to constitute a cell division checkpoint for precise coordination of the divisome activity.

**Fig 9:**
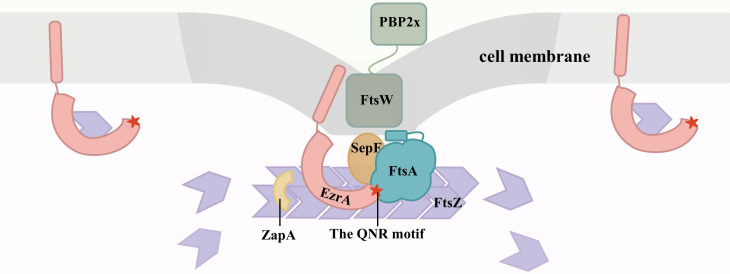
Schematic model depicting the possible mode of action of EzrA in the early stage of Z-ring formation. EzrA (pink) is recruited to the mid-cell point (dark gray) through direct interaction of its QNR motif (red star) with the principal Z-ring membrane tether FtsA (blue-green), and then both of them work collectively to modulate the dynamics of the Z-ring (purple). EzrA and FtsA are also involved in recruiting the late cell division components to the divisome to regulate the assembly of the nascent septal PG with its remodeling by the FtsW/PBP2x pairs (dark green). Moreover, at the cell midpoint, EzrA, SepF (dark orange), and ZapA (yellow) synergistically cooperate to coordinate Z-ring condensation, which is important for septal cell wall constriction initiation. On the other hand, the integral membrane protein EzrA, located at a position other than the division site (light gray), functions to bind the free FtsZ monomer to hamper abnormal Z-ring formation.

In summary, the findings in this study support a model in which FtsA recruits EzrA to the septum via direct interaction with the QNR motif of EzrA to control Z-ring localization. Their persistence at the septum could also synergistically coordinate the recruitment of other divisome components and restrict them to a small region to ensure septation (Fig. 9).

## MATERIALS AND METHODS

### Bacteria strains and growth conditions

The *S. suis* strain SC19 (46) and its derivatives in this study were cultured in tryptic soy broth (TSB; Difco) or on tryptic soy agar (TSA; Difco) plates containing 10% (vol/vol) fetal bovine serum (Every green, China) at 37°C or 28°C. Spectinomycin (100 µg mL^−1^) was used when necessary. The *S. pneumoniae* strains are derived from the capsule-deficient mutant of the D39 strain (*S. pneumonia* D39 Δ*cps rpsL*^+^) (47). Strains from *S. pneumoniae* were routinely grown in C+Y medium (48) or brain heart infusion (BHI; Difco) medium at 37°C in 5% CO_2_ without shaking. Natural transformation of pneumococcal cells was done by adding the synthetic competence-stimulating peptides 1 (CSP 1) and 2 (CSP 2) (49, 50). For growth on the plate, TSA supplemented with 5% (vol/vol) defibrinated sheep blood was used. If appropriate, kanamycin (400 µg mL^−1^), streptomycin (200 µg mL^−1^), or tetracycline (0.5 µg mL^−1^) was used for antibiotic selection. *E. coli* DH5α, BL21 (DE3), and BTH101 were used for plasmid cloning, protein overexpression, and bacteria two-hybrid assay, respectively. *E. coli* strains were grown in LB medium or on LB agar supplemented with spectinomycin (100 µg mL^−1^), kanamycin (50 µg mL^−1^), chloramphenicol (100 µg mL^−1^), or ampicillin (150 µg mL^−1^) when necessary. All strains and plasmids used in this study are listed in Tables S2 and S3. The growth curves were measured using an automatic growth curve analyzer (Oy Growth Curves Ab Ltd., Finland).

### Construction plasmids and bacterial strains

The detailed description of primers related to the construction of the different strains and plasmids is listed in Tables S1 and S2. Unless otherwise noted, all plasmids were constructed using the ClonExpress MultiS one-step cloning kit (Vazyme, Nanjing, China). The plasmid pSET2 is used to indicate the localization of GFP-fused protein forced by the ATc-inducible promoter in *S. suis* (37). The thermosensitive suicide vector pSET4s is used for the mutant construction of *S. suis* (51). pJWV25 plasmid containing a Zn-inducible P_Zn_ promoter is for ectopic gene expression in *S. pneumoniae* (52). pET28a was used for protein expression. The plasmids of pUT18 and pKT25 encoding target proteins were used for bacterial two-hybrid to detect protein-protein interaction(53). All plasmids were confirmed by PCR and Sanger sequencing. *S. suis* mutant strains were generated using the thermosensitive suicide plasmid pSET4s by homologous recombination as previously described (51). *S. pneumoniae* mutant strains were generated using the Janus cassette system (54), which comprised a kanamycin resistance gene and the *rpsL* wild-type gene. The primers used to construct strains and plasmids in this study are listed in Table S4.

### Fluorescence microscopy

Super-resolution fluorescence microscopy was performed to analyze protein subcellular localization, newborn PG, as well as cell morphology using a Nikon structured illumination microscope (Nikon Instruments, Inc.) (55). To determine the subcellular localization of GFP-fused cell division proteins in *S. suis* strains, cells were grown to the early exponential phase (OD_600_ of 0.2), diluted into fresh TSB medium with a ratio of 25:1, and grown for 45 min. Afterward, protein expression was induced by adding ATc at the appropriate final concentration and incubation time (50 ng/mL, 10 min for FtsZ-GFP; 50 ng/mL, 6 min for GFP-FtsA; 50 ng/mL, 10 min for SepF-GFP; 60 ng/mL, 10 min for ZapA-GFP). The cells were then stained with other fluorescent dyes to indicate newborn PG or cell outline, followed by SIM analysis. For fluorescence microscopic analysis of the subcellular localization of GFP-fused proteins in *S. pneumoniae*, exponentially growing cells were diluted with fresh BHI medium to an OD_600_ of 0.01, followed by incubation at 37°C for 3 h without shaking. Then, 0.4 mM ZnCl_2_ and 0.04 mM MnSO_4_ were added to induce protein expression, followed by incubation at 37°C for 3 h without shaking. The cells were then stained with other fluorescent dyes to indicate newborn PG or cell outline, followed by SIM analysis. To label newborn PG, cells were stained with fluorescent D-amino acid (HADA) (WuXi App Tec) at a final concentration of 250 µM for 10 min at 37°C with shaking for *S. suis*, and for 20 min at 37°C in 5% CO_2_ without shaking for *S. pneumoniae*. The cells were then washed three times with phosphate-buffered saline (PBS). When applicable, dynamic PG synthesis of *S. suis* cells was determined by sequentially pulse-labeling the growing cells with two fluorescent D-amino acids of different colors, a long pulse of HADA staining (20 min) followed by a short pulse of TADA staining (30 s). To indicate the cell outline, the *S. suis* and *S. pneumoniae* strains were stained with fluorescent dye Alexa Fluor 647 NHS ester (AF647; Thermo Fisher Scientific). Briefly, cells were treated with AF647 at a final concentration of 20 µg/mL for 45 min at 37°C without shaking and washed with PBS. Then pneumococcal cells required an exceptional fixation procedure: cells were fixed in the dark and incubated with 500 µL 4% polyoxymethylene for 15 min at room temperature, followed by 45 min at 4°C and washed with PBS (56). Finally, the stained cells were spotted on a slide and covered with a 0.08% agarose pad for imaging by SIM. GFP-fused proteins were imaged with excitation at 488 nm and emission at 500 nm to 545 nm. The TADA-labelled cells or mCherry-fused proteins were imaged with excitation at 561 nm and emission at 570 nm to 640 nm. The HADA-labeled cells were imaged with excitation at 405 nm and emission at 435 nm to 545 nm. AF647-labeled cells were imaged with excitation at 647 nm and emission at 663 nm to 738 nm.

### Transmission electron microscopy

*S. suis* cells grown to the exponential phase were fixed overnight with 2.5% glutaraldehyde at 4°C. The cells were refixed with 1% osmic acid at room temperature for 2 h. Successive ethanol gradient dehydration (50% vol/vol, 75% vol/vol, 95% vol/vol, and 100% vol/vol ethanol) was performed for 15 min. The dehydrated cells were infiltrated in acetone and embedded in an epoxy resin. Thin sections (80 nm) were generated by ultramicrotome and loaded onto 150-mesh copper grids, followed by positive staining with 2% uranium acetate saturated alcohol solution for 8 min and rinsing several times with 70% ethanol and ddH_2_O. An H-7650 transmission electron microscope (Hitachi, Japan) with an accelerating voltage of 80 kV was used for imaging.

### Scanning electron microscopy

*S. suis* cells grown to the exponential phase were washed three times with PBS. The cell suspension was spotted on glass coverslips and fixed in 2.5% glutaraldehyde overnight at 4°C. The fixed samples were then dehydrated with a series of gradient ethanol (30%, 50%, 70%, 100%) for 15 min, respectively, air-dried, and finally covered with a 10 nm-thick gold/platinum layer (JSM-6390LV, JEOL, Japan). Samples were then observed with a scanning electron microscope (JFC-1600, JEOL, Tokyo, Japan).

### Western blotting

The cell pellets of *S. suis* or *E. coli* cultures were washed twice with PBS. Then the samples were resuspended in lysis buffer (0.1 M Tris-HCl, pH 7.5, 150 mM NaCl, 1% Triton X-100, 0.2 mg/mL protease inhibitor, 500 U/µL mutanolysin) and treated by sonication. The entire process was rigorously performed on ice. After sonication, the supernatant was collected by centrifugation, and the protein concentrations were adjusted to the same level. Then the samples added with loading buffer were resolved on 12% SDS-polyacrylamide gel and transferred onto polyvinylidene fluoride membrane (Millipore) using a Transfer Device (Bio-Rad). The membrane was blocked with 5% non-fat milk in Tris-Buffered Saline (TBS: 50 mM Tris, pH 7.5, 150 mM NaCl) containing 0.05% Tween-20 overnight at 4°C. The EzrA protein and its mutants were detected using EzrA polyclonal antibody at a 1:1,000 dilution. The antibody was produced by immunized mice twice with the purified recombinant EzrA protein expressed in *E. coli*. Horseradish Peroxidase (HRP) conjugated goat anti-mouse IgG (Proteintech, Wuhan, China) was used as the secondary antibody at a 1:5,000 dilution. Signal detection was performed by using the ECL substrate and chemiluminescence imaging system.

### Co-immunoprecipitation and mass spectrometry

*S. suis* cells with or without 3×FLAG-tagged protein were grown at 37°C in TSB until the OD_600_ of 0.1 was reached. The cells were pelleted and resuspended in cold lysis buffer (0.1 M Tris-HCl, pH 7.5, 150 mM NaCl, 1% Triton X-100, 0.2 mg/mL protease inhibitor [Sigma] containing 500 U/µL mutanolysin). The cells were then incubated for 45 min at 37°C and disrupted by sonication on ice. The cell lysate was normalized to a total protein of 1 mg and then incubated with 50 µl FLAG magnetic agarose slurry (Thermo Scientific) for 20 min at room temperature. Protein-bound beads were washed three times with PBS and eluted in 4 × Laemmli buffer at 90°C for 5 min. The eluted sample was subjected to mass spectrometry as well as analyzed by SDS-PAGE and immunoblotting using an anti-FLAG antibody. Mass spectrometry analysis was carried out using a Q Exactive Plus LC/MS system (Thermo Scientific). The mass spectral data produced by the Q Exactive Plus were then analyzed using MaxQuant (v.1.6.2.10) with the MaxLFQ search algorithm. The Proteomic Reference Database of *S. suis* in UniProt was used for database search.

### Bacterial two-hybrid assay

The B2H experiments were performed to determine protein-protein interaction according to the manufacturer’s instructions and reference (53). Plasmids of pUT18 and pKT25 encoding the respective genes were co-transformed into the *E. coli* BTH101 reporter cells. Fresh colony of the co-transformant was picked and resuspended in 200 µL of LB, and 3 µL of the cell suspension was spotted onto LB agar plates containing chloramphenicol (50 µg mL^−1^), ampicillin (100 µg mL^−1^), streptomycin (100 µg mL^−1^), X-Gal (40 µg mL^−1^), and IPTG (0.5 mM), followed by incubated at 30°C in the dark for 24 h. The plate was then imaged using a digital camera. Plasmid pairs pKT25/pUT18 and pKT25-zip/pUT18C-zip were taken as negative and positive control, respectively. Quantitative determination of the B2H results was achieved by measuring the activity of β-galactosidase as previously described (55). Briefly, individual colonies from different co-transformants harboring pUT18- and pKT25-derived plasmids were cultured at 30°C for 16 h. The cells were then diluted with M63 medium in 96-well plates, and the OD_600_ was measured. Then cells were permeabilized with incubation of 0.05% SDS and chloroform. The cell lysate was mixed with PM2 medium and incubated at room temperature for 10 to 20 min. The stop solution containing 1 M Na_2_CO_3_ was added to terminate the reaction until the medium color turned yellow. A microplate reader was used to measure the absorbance at 405 nm (OD_405_). The enzyme activity is calculated as follows: relative units = 1,000 × ((OD_405_ in sample wells − OD_405_ in control wells) / (OD_600_ in sample wells − OD_600_ in control wells)) / time (min) of incubation).

### Protein purification

The his-tagged proteins were expressed using the pET28a plasmid in *E. coli* BL21 (DE3) cells and purified as previously described (27). Briefly, the cytoplasmic region of EzrA protein of *S. suis* (EzrA_cyto_) and its variants were C-terminally fused with a SUMO tag. The overexpression of His-SUMO-tagged proteins was induced with 0.5 mM IPTG, at 25°C for 10 h. The cells were harvested and resuspended in lysis buffer (50 mM Tris-HCl, pH 8.0, 300 mM NaCl, 10 mM imidazole) and lysed by a French press. The lysate supernatant was purified using chromatography with a Ni-NTA column (HUIYAN Bio, China). The His-SUMO tag was then cleaved by incubation with the SUMO protease for 16 h at 4°C and removed by incubation with Ni-NTA resin. The cleaved protein was then exchanged with storage buffer (2 mM Tris-HCl, pH 8.0, 10 mM NaCl), concentrated, snap-frozen in liquid nitrogen, and stored at 80°C. The his-tagged FtsA protein was purified in the same way but without the SUMO cleavage step.

### Surface plasmon resonance

SPR assay was carried out using a Biacore T200 system. Briefly, FtsA protein dissolved in binding buffer was covalently immobilized onto a CM5 sensor chip surface to reach 700 response units, and then all the channels were quenched by 1 M ethanolamine. The uncoated channel was used as a reference control. Increasing concentrations in twofold dilutions (from 200 µM to 3.125 µM) of EzrA_cyto_ or its variant in binding buffer was injected at a flow rate of 30 µL min^−1^ at 25°C. The fitting sensorgrams were generated using Biacore T200 Evaluation Software with a 1:1 binding model.

### Protein alignment

The EzrA protein sequences were aligned using Clustal Omega, and the alignment was presented using Jalview with the conserved motif highlighted (57, 58).

### Image analysis

The fluorescent intensity of interest regions was measured using ImageJ/Fiji software and plotted by Origin software. Cell septum relative position, cell length and width imaged by NIS-Elements (Nikon), and septum thickness imaged by TEM were measured with ImageJ and the ObjectJ plugin and plotted by GraphPad Prism version 9.5.

### Statistic and reproducibility

GraphPad Prism version 9.5 (GraphPad Software, Boston, MA, USA) was applied to analyze statistical significance between two or more groups. Statistical analysis between two groups was determined by the Mann-Whitney U-test. Statistical analysis for groups of more than two was determined by one-way analysis of variance.

## SUPPLEMENTAL MATERIAL

Figures S1 to S7 and Tables S1 to S4. jb.00125-25-s0001.docx Fig. S1. Validation of the EzrA-mCherry fusion in *S. suis*. Fig. S2. Validation of the CΔezrA of *S. suis*. Fig. S3. TEM micrographs of *S. suis* cells of strains SC19, ΔezrA, ezrAR514D and ezrAΔQNR. Fig. S4. Determination of the targets that interact with the QNR motif of EzrA through the bacteria two-hybrid screen. Fig. S5. Purification of FtsA, EzrAcyto, and EzrAR514Dcyto. Fig. S6. Validation of the mCherry-spnFtsA fusion and the PZn-mCherry-spnFtsA fusion in *S. pneumoniae*. Fig. S7. Validation of the spnEzrA-GFP fusion in *S. pneumoniae*. Table S1. Cell division proteins with differential abundance co-immunoprecipitated with EzrA and EzrAR514D. Table S2. Strains used in this study. Table S3. Plasmids used in this study. Table S4. Oligonucleotide primers used in this study.

ASM does not own the copyrights to Supplemental Material that may be linked to, or accessed through, an article. The authors have granted ASM a non-exclusive, world-wide license to publish the Supplemental Material files. Please contact the corresponding author directly for reuse.
