# Supplementary material for: EzrA promotes Z-ring formation through interaction of its QNR motif with FtsA
Source: J Bacteriol. 2025 Jul 3;207(7):e00125-25. doi: 10.1128/jb.00125-25 (PMC12288466; doi:10.1128/jb.00125-25)
Supplement: Figures S1 to S7 and Tables S1 to S4. — Fig. S1. Validation of the EzrA-mCherry fusion in S. suis. Fig. S2. Validation of the CΔezrA of S. suis. Fig. S3. TEM micrographs of S. suis cells of strains SC19, ΔezrA, ezrAR514D and ezrAΔQNR. Fig. S4. Determination of the targets that interact with the QNR motif of EzrA through the bacteria two-hybrid screen. Fig. S5. Purification of FtsA, EzrAcyto, and EzrAR514Dcyto. Fig. S6. Validation of the mCherry-spnFtsA fusion and the PZn-mCherry-spnFtsA fusion in S. pneumoniae. Fig. S7. Validation of the spnEzrA-GFP fusion in S. pneumoniae. Table S1. Cell division proteins with differential abundance co-immunoprecipitated with EzrA and EzrAR514D. Table S2. Strains used in this study. Table S3. Plasmids used in this study. Table S4. Oligonucleotide primers used in this study. [file jb.00125-25-s0001.docx]

**SUPPLEMENTAL MATERIALS**

**
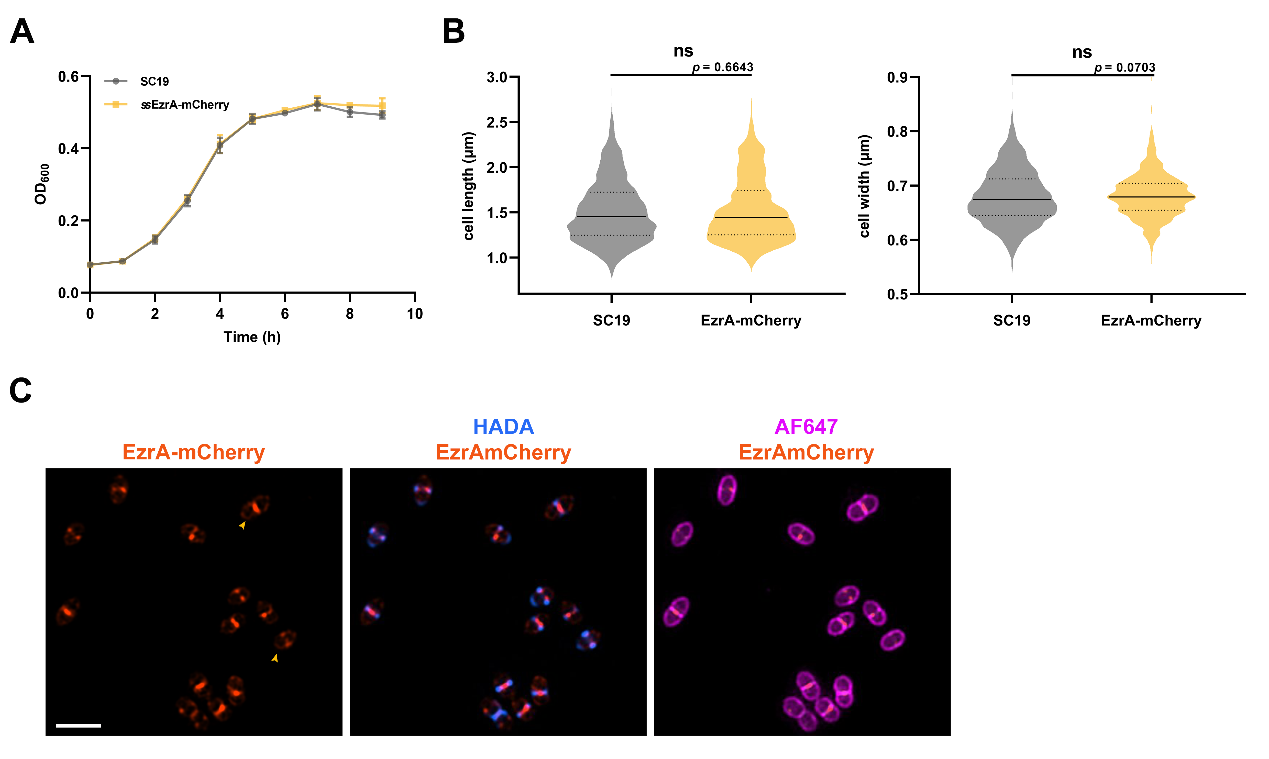
**

**Figure S1. Validation of the EzrA-mCherry fusion in *S. suis*. (A)** Growth curves of SC19 and EzrA-mCherry strains. The cells of each indicated strain were inoculated into TSB medium from overnight grown culture and then incubated at 37°C with shaking. **(B)** Violin plots displaying the distribution of the cell length (left panel) and cell width (right panel) for *S. suis* SC19 (gray) and EzrA-mCherry (yellow) as analyzed using ObjectJ. The dot lines indicate the 25th and 75th percentile and the median is indicated with a line. Statistical analysis was done using Mann Whitney test, ns indicates no significant difference. From left to right: n = 1075 and 1037 cells. **(C)** Subcellular localization pattern of EzrA-mCherry. The cells were grown to the mid-log phase. The cells were stained with HADA for 10 min followed by staining with AF647 and then imaged by SIM. The yellow arrows indicate the slightly localized EzrA-mCherry at the cell membrane. Scale bar, 2 µm.


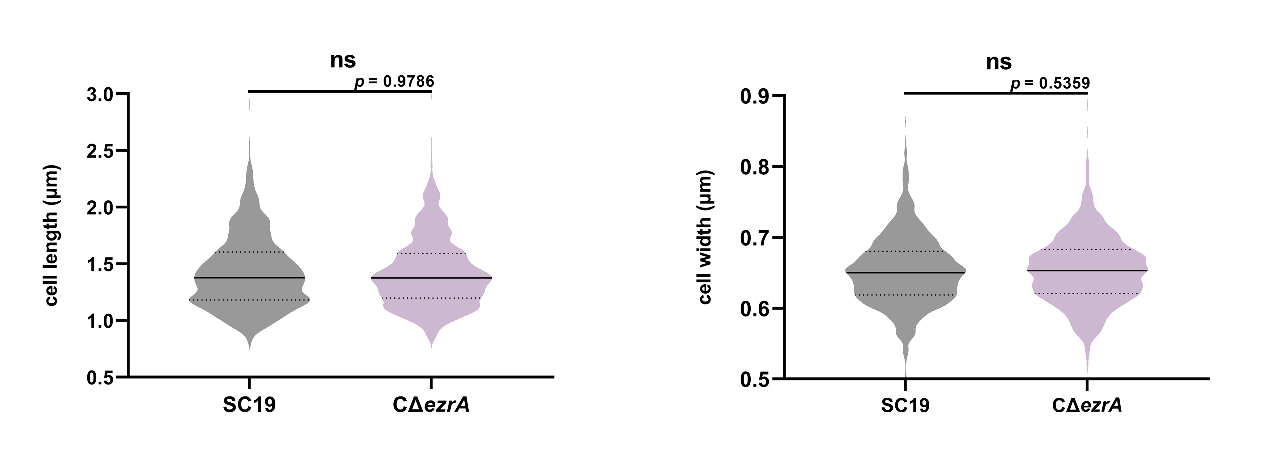


**Figure S2. Validation of the CΔ*ezrA* of *S. suis*.** Violin plots displaying the distribution of the cell length (left panel) and cell width (right panel) for *S. suis* SC19 (gray) and CΔ*ezrA* (purple) as analyzed using ObjectJ. The dot lines indicate the 25th and 75th percentile, and the median is indicated with a line. Statistical analysis was done using Mann Whitney test, and ns indicates no significant difference. From left to right: n = 837 and 863 cells.


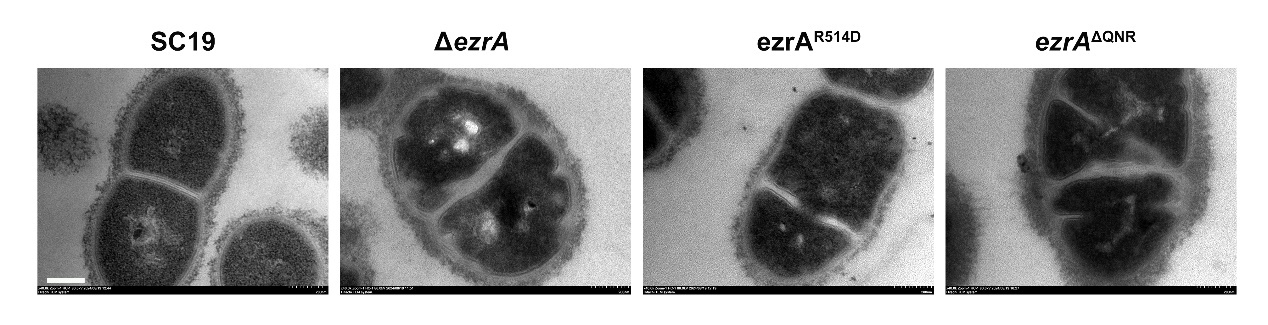


**Figure** **S3. TEM micrographs of *S. suis* cells of strains SC19, Δ*ezrA*, *ezrA*^R514D^ and *ezrA*^ΔQNR^.** Mutations on EzrA induced a significantly thicker septal cell wall compared to SC19 cells. The cells of each indicated strain were grown to the mid-cell phase and analyzed by TEM. Scale bar, 200 nm.

**
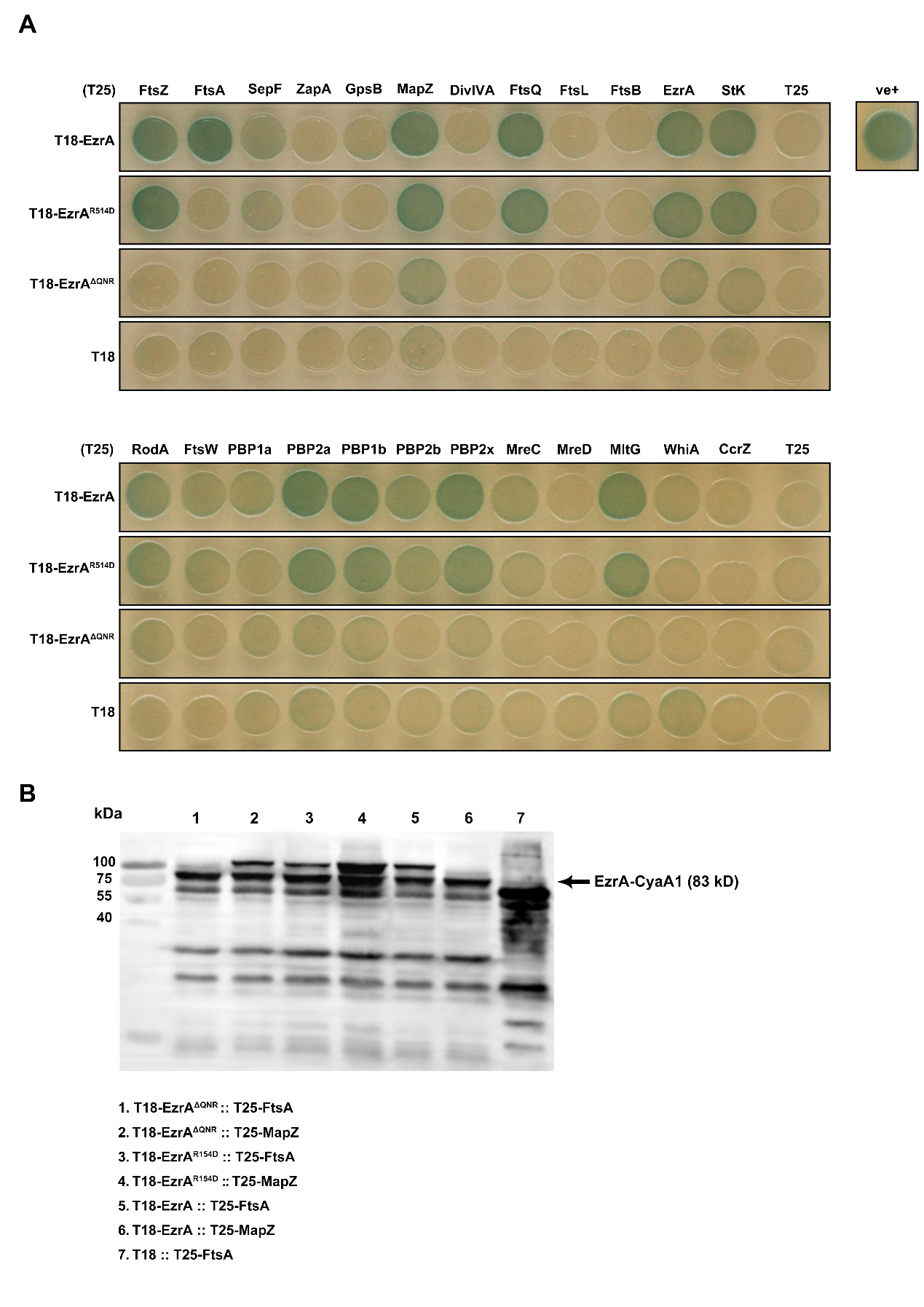
**

**Figure** **S4. Determination of the targets that interact with the QNR motif of EzrA through the bacteria two-hybrid screen.** **(A)** Bacterial two-hybrid assay. The primary divisome components fused to pKT25 (upper panel) and the PG synthesis-related proteins fused to pKT25 (under panel) were selected to co-transform with pUT18-EzrA, pUT18-EzrA^R514D^, and pUT18-EzrA^ΔQNR^, respectively. The interaction pair of pUT18-zip and pKT25-zip was used as a positive control. *E. coli* BTH101 cells expressing each indicated T18- and T25-fused proteins were spotted onto LB agar plates containing chloramphenicol, ampicillin, streptomycin, X-Gal, and IPTG, followed by incubation at 30°C in dark for 24 h. **(B)** Western immunoblot of whole-cell lysates from *E. coli* BTH101 harboring pUT18- and pKT25-derived plasmids as described in *Material* and *Methods*. Cells were grown to exponential phase in the presence of 0.5 M IPTG at 30 ℃ for 6 h. The EzrA antibody was used to determine the presence of CyaA-fused EzrA or its mutants.


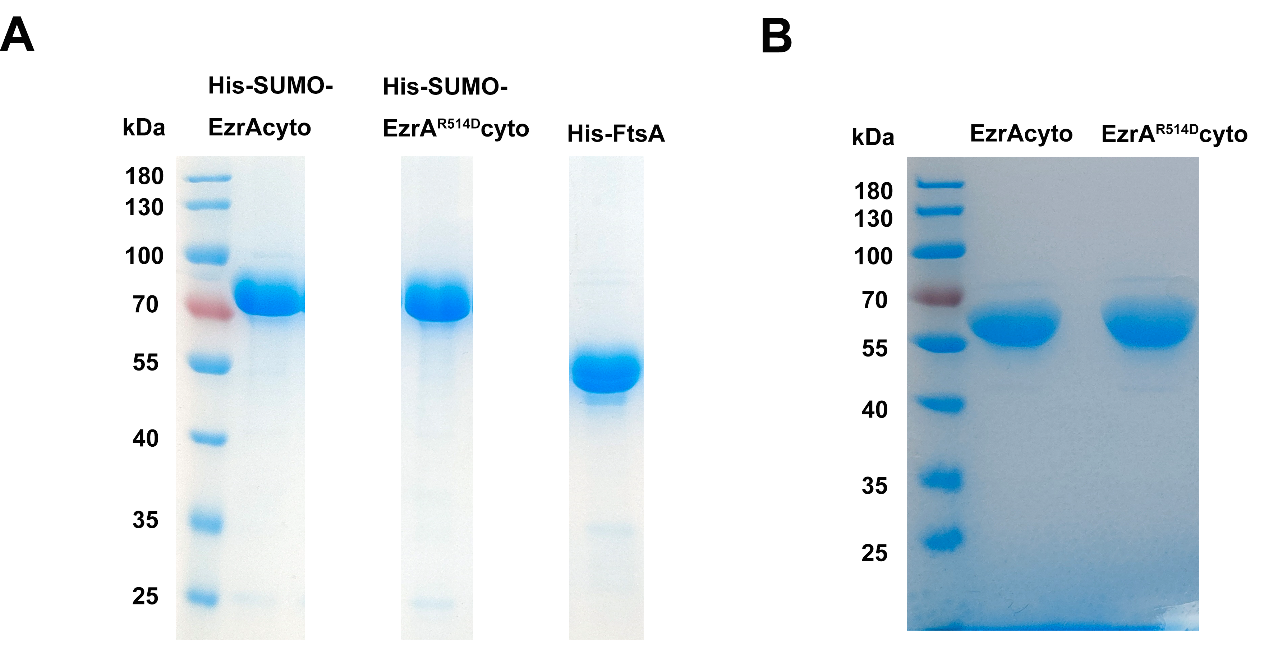


**Figure** **S5. Purification of FtsA, EzrAcyto, and EzrA^R514D^cyto.** **(A)** Protein purification. Proteins were overproduced in *E. coli* BL21 (DE3) as His-tagged fusion of FtsA and His-SUMO-tagged fusion of EzrAcyto and EzrA^R514D^cyto. After Ni-NTA affinity purification, proteins were analyzed using SDS-PAGE. **(B)** To avoid the interference of SUMO to the intrinsic structure of EzrA, the His-SUMO tag was cleaved by SUMO protease and removed by Ni-NTA resin. The untagged EzrAcyto and EzrA^R514D^cyto were analyzed using SDS-PAGE.


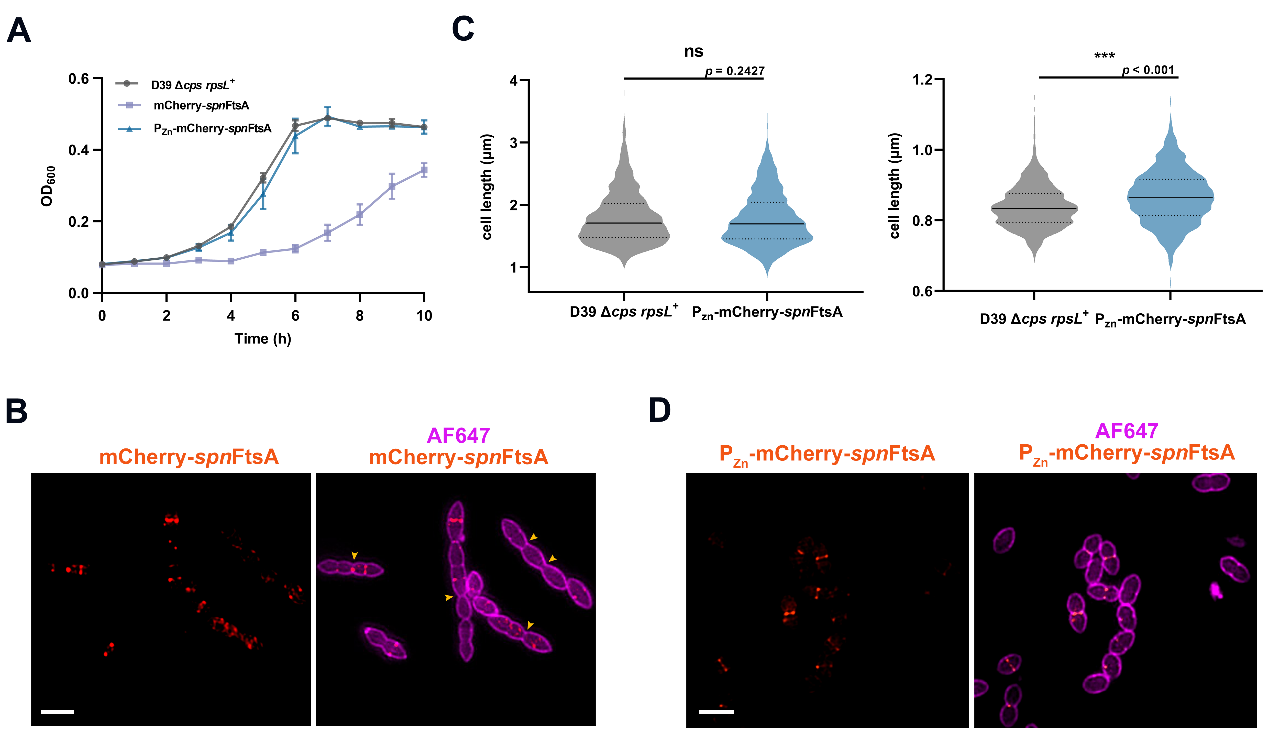


**Figure** **S6. Validation of the mCherry-*spn*FtsA fusion and the P_Zn_-mCherry-*spn*FtsA fusion in *S. pneumoniae*. (A)** Growth curves of the pneumococcal strains of D39 Δ*cps rpsL*^+^ and mCherry-*spn*FtsA, and P_Zn_-mCherry-*spn*FtsA. The cells of each indicated strain were inoculated into BHI medium from overnight grown culture and then incubated at 37°C in 5% CO_2_ without shaking. **(B)** Subcellular localization pattern of natively chromosomal-expressing mCherry-FtsA. The cells were grown to the mid-log phase. The cells were stained with AF647 and imaged by SIM. The yellow arrows indicate the abnormally undivided cells with elongated cell shape. Scale bar, 2 µm. **(C)** Violin plots displaying the distribution of the cell length (left panel) and cell width (right panel) for D39 Δ*cps rpsL* (gray) and P_Zn_-mCherry-*spn*FtsA (blue) strains as analyzed using ObjectJ. The dot lines indicate the 25th and 75th percentile and the median is indicated with a line. Statistical analysis was achieved using Mann Whitney test, *** *p*< 0.001, and ns indicates no significant difference. From left to right: n = 1288 and 1276 cells. **(D)** Subcellular localization pattern of chromosomal-expressing mCherry-FtsA (Zn^2+^ inducible). The cells were grown to the mid-log phase. The cells were stained with AF647 and imaged by SIM. mCherry-*spn*FtsA localizes correctly at the mid-cell, although cells showing widen morphology (C, right panel). Scale bar, 2 µm.

**
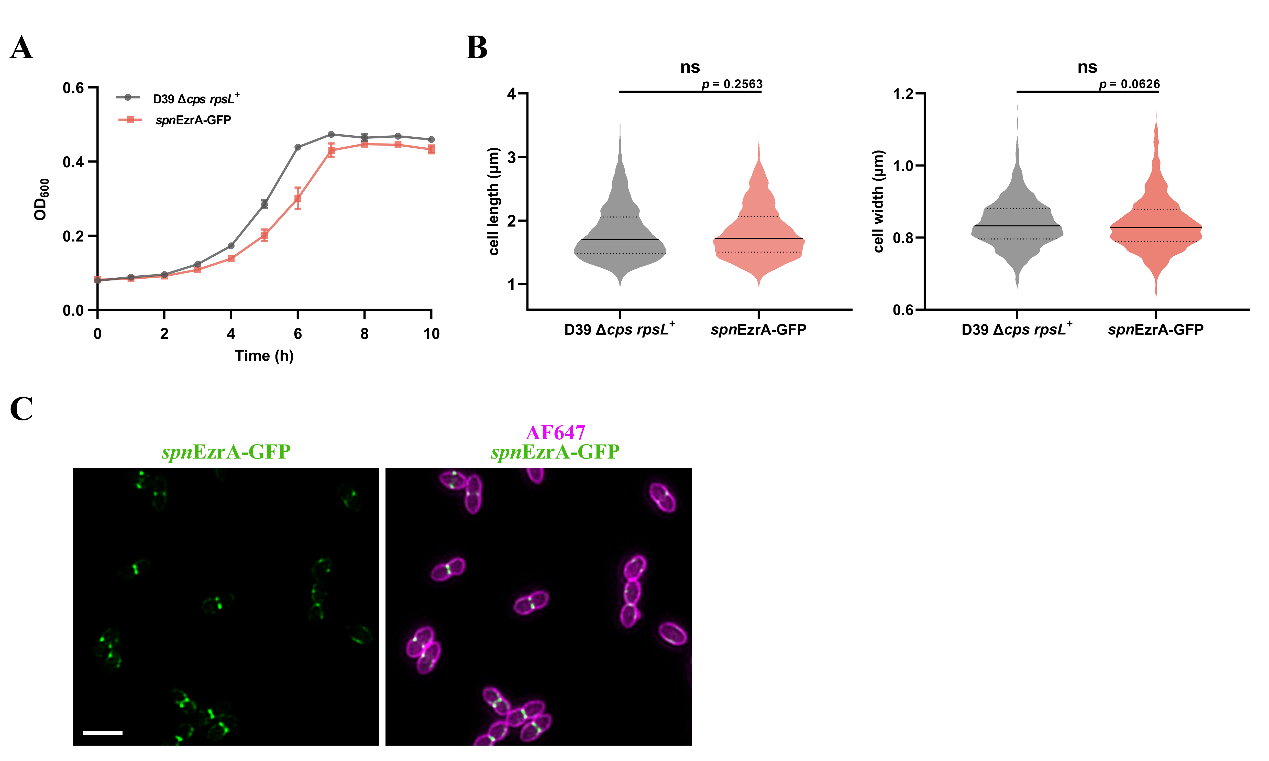
**

**Figure** **S7. Validation of the *spn*EzrA-GFP fusion in *S. pneumoniae*.** (A) Growth curves of the pneumococcal strains of D39Δ*cps rpsL*^+^ and *spn*EzrA-GFP. The cells of each indicated strain were inoculated into BHI medium from overnight grown culture and then incubated at 37°C in 5% CO_2_ without shaking. (B) Violin plots displaying the distribution of the cell length (left panel) and cell width (right panel) for D39 Δ*cps rpsL* (gray) and *spn*EzrA-GFP (pink) strains as analyzed using ObjectJ. The dot lines indicate the 25th and 75th percentile and the median is indicated with a line. Statistical analysis was achieved using Mann Whitney test. ns indicates no significant difference. From left to right: n = 1226 and 1285 cells. (C) Subcellular localization pattern of chromosomal-expression *spn*EzrA-GFP. The cells were grown to the mid-log phase. The cells were stained with AF647 and imaged by SIM. Scale bar, 2 µm.

**Table 1. Cell division proteins with differential abundance co-immunoprecipitated with EzrA and EzrA^R514D^ ^a^**

| **Protein** | **Locus** | **Function** | **Molecular mass (kDa)** | **EzrA^R514D^ /**  **EzrA** | |
| --- | --- | --- | --- | --- | --- |
|  |  |  |  | **Log_2_ FC ^b^** | **Diff**  **Sig ^c^** |
| EzrA | SSU05_1509 | Z-ring formation regulator | 66.43 | +0.1827 | NA |
| DivIC | SSU05_0010 | Membrane of DivIC/FtsL/DivIB complex which function to regulate septal PG synthesis | 14.43 | -0.9554 | - |
| FtsL | SSU05_1743 | Membrane of DivIC/FtsL/DivIB complex which function to regulate septal PG synthesis | 12.03 | -0.8757 | - |
| DivIB | SSU05_0478 | Membrane of DivIC/FtsL/DivIB complex which function to regulate septal PG synthesis | 40.13 | -0.6759 | - |
| PBP2x | SSU05_1742 | Membrane of the divisome machinery that synthesizes the septal cross wall | 82.63 | -0.5393 | - |
| MltG | SSU05_1718 | Membrane-bound peptidoglycan glycosidase, role in cleaving nascent peptidoglycan strands | 67.1 | -0.5131 | - |
| RodA | SSU05_1512 | Lipid II flippase; role in peripheral PG synthesis | 45.63 | -0.4311 | - |
| GpsB | SSU05_0417 | Cell cycle protein that functions to regulating regulate septal PG synthesis | 12.86 | -0.4182 | - |
| DivIVA | SSU05_0487 | membrane-associated coiled-coil protein that required for completing of cell division and separation | 26.21 | -0.3754 | - |
| MreC | SSU05_0019 | Maintenance of rod shape, regulation of peptidoglycan synthesis by the elongasome | 29.92 | -0.3743 | - |
| PBP2a | SSU05_1985 | Class A PBPs, role in remodel the nascent PG mesh | 81.54 | -0.2757 | - |
| PBP1b | SSU05_0120 | Class A PBPs, role in remodel the nascent PG mesh | 87.23 | -0.2323 | - |
| FtsE | SSU05_1411 | Part of the ABC transporter FtsEX involved in controlling periplasmic PG hydrolase activities | 27.7 | -0.1631 | NA |
| MapZ | SSU05_0419 | Mid-cell-anchored protein that marks the future division site | 52.5 | -0.1072 | NA |
| PBP2b | SSU05_1354 | Membrane of the elongasome machinery that synthesizes the peripheral cross wall | 75.3 | -0.0075 | NA |
| FtsX | SSU05_1410 | Part of the ABC transporter FtsEX involved in controlling periplasmic PG hydrolase activities | 35.4 | 0.0197 | NA |
| PBP1a | SSU05_0414 | Class A PBPs, role in remodel the nascent PG mesh | 79.4 | 0.1967 | NA |

^a^ The co-immunoprecipitation experiment was performed as described in the “Materials and methods”.

^b^ The log_2_ FC describes the differences of LFQ intensity immunoprecipitated from the cells expressing EzrA^R514D^ to those expressing EzrA (control).

^c^ Difference Significance proteins were determined according to the Log2 FC values. The value (>0.263) indicates upregulated protein to form complex with EzrA^R514D^ compared to EzrA and uses a “+” to denote, while the value (-0.263) indicates downregulated protein in the complex of EzrA^R514D^ and uses a “-” to denote. “NA” indicates proteins with no detected difference.

**Table S2. Strains used in this study**

| **Strains** | **Description** | **Reference or source** |
| --- | --- | --- |
| *Escherichia coli* |  | Lab stock |
| DH5α | F*-*, 80*dlacZ* M15 *(lacZYA-argF)* U169 *recA1 endA1hsdR17(rk-, mk+) phoAsupE44 -thi-1 gyrA96 relA1; for cloning* | Lab stock |
| BL21 (DE3) | *F-, ompT gal dcm lon hsdSB (rB-mB-) λ (DE3 [lacI lacUV5-T7 gene 1 ind1 sam7 nin5]);* *for expression* | Lab stock |
| BTH101 | *F-, cya-99, araD139, galE15, galK16, rpsL1 (StrR), hsdR2, mcrA1, mcrB1; for expression* | (1) |
| *Streptococcus suis* | | |
| SC19 | Clinical isolate, wild type | (2) |
| Δ*ezrA* | SC19, Δ*ezrA* | This study |
| CΔ*ezrA* | SC19, Δ*ezrA* + *ezrA* | This study |
| *ezrA*^R514D^ | SC19, *ezrA*^R514D^ | This study |
| *ezrA*^ΔQNR^ | SC19, *ezrA*^ΔQNR^ | This study |
| EzrA-mCherry | SC19, *ezrA-mCherry* | This study |
| EzrA^R514D^-mCherry | SC19, *ezrA*^R514D^*-mCherry* | This study |
| EzrA^ΔQNR^-mCherry | SC19, *ezrA*^ΔQNR^*-mCherry* | This study |
| EzrA-mCherry-P_tetR_-FtsZ-GFP | SC19, *ezrA-mCherry,* P_tetR_-*ftsZ-gfp* with pSET2; Spc^R^ | This study |
| P_tetR_-FtsZ-GFP | SC19, P_tetR_-*ftsZ-gfp* with pSET2; Spc^R^ | This study |
| Δ*ezrA*-P_tetR_-FtsZ-GFP | SC19, Δ*ezrA,* P_tetR_-*ftsZ-gfp* with pSET2; Spc^R^ | This study |
| EzrA-3xFLAG | SC19, *ezrA-3xflag* | This study |
| EzrA^R514D^-3xFLAG | SC19, *ezrA*^R514D^*-3xflag* | This study |
| P_tetR_-GFP-FtsA | SC19, P_tetR_*- gfp-ftsA* with pSET2; Spc^R^ | This study |
| P_tetR_-SepF-GFP | SC19, P_tetR_-*sepF-gfp* with pSET2; Spc^R^ | This study |
| P_tetR_-ZapA-GFP | SC19, P_tetR_*-zapA-gfp* with pSET2; Spc^R^ | This study |
| Δ*ezrA*-P_tetR_-GFP-FtsA | SC19, Δ*ezrA,* P_tetR_*- gfp-ftsA* with pSET2; Spc^R^ | This study |
| Δ*ezrA*-P_tetR_-SepF-GFP | SC19, Δ*ezrA,* P_tetR_-*sepF-gfp* with pSET2; Spc^R^ | This study |
| Δ*ezrA*-P_tetR_-ZapA-GFP | SC19, Δ*ezrA,* P_tetR_*-zapA-gfp* with pSET2; Spc^R^ | This study |
| *ezrA*^R514D^-P_tetR_-FtsZ-GFP | SC19, *ezrA*^R514D^*,* P_tetR_-*ftsZ-gfp* with pSET2; Spc^R^ | This study |
| *ezrA*^R514D^-P_tetR_-GFP-FtsA | SC19, *ezrA*^R514D^*,* P_tetR_*- gfp-ftsA* with pSET2; Spc^R^ | This study |
| *ezrA*^R514D^-P_tetR_-SepF-GFP | SC19, *ezrA*^R514D^*,* P_tetR_-*sepF-gfp* with pSET2; Spc^R^ | This study |
| *ezrA*^R514D^-P_tetR_-ZapA-GFP | SC19, *ezrA*^R514D^*,* P_tetR_*-zapA-gfp* with pSET2; Spc^R^ | This study |
| EzrA-mCherry-P_tetR_-GFP-FtsA | SC19, *ezrA-mCherry,* P_tetR_*-gfp-ftsA* with pSET2; Spc^R^ | This study |
| EzrA^R514D^-mCherry-P_tetR_-GFP-FtsA | SC19, *ezrA*^R514D^*-mCherry,* P_tetR_*-gfp-ftsA* with pSET2; Spc^R^ | This study |
| *Streptococcus pneumoniae* | |  |
| D39 Δ*cps rpsL*^+^ | D39 Δ*cps rpsL*^+^*; Str^R^* | (3) |
| Janus::*spn*FtsA | D39 Δ*cps rpsL*^+^, *P_c_-*[*kan-rpsl*]-*ftsA*; *kan^R^* | (4) |
| mCherry-*spn*FtsA | D39 Δ*cps rpsL*^+^, *mCherry-ftsA*; *Str^R^* | This study |
| *spn*EzrA-GFP::Janus | D39 Δ*cps rpsL*^+^, *ezrA*-*gfp*-*P_c_*-[*kan-rpsl*]; *kan^R^* | This study |
| *spn*EzrA-GFP | D39 Δ*cps rpsL*^+^, *ezrA*-*gfp*; *Str^R^* | This study |
| *spn*EzrA^QND^-GFP::Janus | D39 Δ*cps rpsL*^+^, *ezrA*^QND^-*gfp-P_c_*-[*kan-rpsl*]; *kan^R^* | This study |
| *spn*EzrA^QND^-GFP | D39 Δ*cps rpsL*^+^, *ezrA*^QND^*-gfp*; *Str^R^* | This study |
| *spn*EzrA-GFP-P_Zn_-mCherry-*spn*FtsA | D39 Δ*cps rpsL*^+^, *ezrA-gfp*, *bgaA*::*P_Zn_-mCherry-ftsA*; *Str^R^ Tet^R^* | This study |
| *spn*EzrA^QND^-GFP-P_Zn_-mCherry-*spn*FtsA | D39 Δ*cps rpsL*^+^, *ezrA*^QND^*-gfp*, *bgaA::P_Zn_-mCherry-ftsA*; *Str^R^ Tet^R^* | This study |
| **Other species** | | |
| *Enterococcus faecalis* ATCC29212 | Clinical isolated, wild type | Lab stock |
| *Bacillus. subtilis* WB800N | Clinical isolated, wild type | Lab stock |
| *Staphylococcus aureus* RN4220 | Clinical isolated, wild type | Lab stock |

**Table S3. Plasmids used in this study**

| Plasmids | Description | Reference or source |
| --- | --- | --- |
| pSET4s | *S. suis* suicide vector for gene deletions and insertions; Spc^R^ | (5) |
| pSET2 | *S. suis* expression vector; Spc^R^ | (5) |
| pSSTete2-gfp | pSET2 derivative containing *gfp* under control of ATc-inducible P_tetR_ promoter; Spc^R^ | (6) |
| pET28a | *E. coli* protein expression vector; Kan^R^ | Lab stock |
| pET28a-SUMO | *E. coli* protein expression vector; Kan^R^ | Lab stock |
| pUT18 | P_lac_-T18-; Amp^R^ | (1) |
| pUT18C | P_lac_-T18-; Amp^R^ | (1) |
| pKT25 | P_lac_-T25-; Chl^R^ | (1) |
| pJWV25 | *S. pneumoniae* expression vector containing P_Zn_ promoter and GFP; Amp^R^, Tet^R^ | (1) |
| pSET4s-Δ*ezrA* | pSET4s derivative containing up-and downstream of *ezrA*; Spc^R^ | This study |
| pSET4s-CΔ*ezrA* | pSET4s derivative containing up-and downstream of *ezrA* and full-length of *ezrA*; Spc^R^ | This study |
| pSET4s-*ezrA*^R514D^ | pSET4s derivative containing up-and downstream of *ezrA*^R514D^; Spc^R^ | This study |
| pSET4s-*ezrA^ΔQNR^* | pSET4s derivative containing up-and downstream of *ezrA^ΔQNR^*; Spc^R^ | This study |
| pSET4s-EzrA-mCherry | pSET4s derivative containing an *ezrA-mCherry* and the downstream region of *ezrA*; Spc^R^ | This study |
| pSET4s-ezrA^R514D^-mCherry | pSET4s derivative containing an *ezrA*^R514D^*-mCherry* and the downstream region of *ezrA*; Spc^R^ | This study |
| pSET4s-ezrA^ΔQNR^-mCherry | pSET4s derivative containing an *ezrA*^ΔQNR^*-mCherry* and the downstream region of *ezrA*; Spc^R^ | This study |
| pSET4s-EzrA-3xFLAG | pSET4s derivative containing an *ezrA-3xflag* and the downstream region of *ezrA*; Spc^R^ | This study |
| pSET4s-EzrA^R514D^-3xFLAG | pSET4s derivative containing an *ezrA*^R514D^*-3xflag* and the downstream region of *ezrA*; Spc^R^ | This study |
| pSET2- P_tetR_-FtsZ-GFP | pSET2 derivative containing *ftsZ-gfp* fusion under control of ATc-inducible P_tetR_ promoter; Spc^R^ | This study |
| pSET2 - P_tetR_-GFP-FtsA | pSET2 derivative containing *gfp-ftsA* fusion under control of ATc-inducible P_tetR_ promoter; Spc^R^ | This study |
| pSET2-P_tetR_-SepF-GFP | pSET2 derivative containing *sepF-gfp* fusion under control of ATc-inducible P_tetR_ promoter; Spc^R^ | This study |
| pSET2-P_tetR_-ZapA-GFP | pSET2 derivative containing *zapA-gfp* fusion under control of ATc-inducible P_tetR_ promoter; Spc^R^ | This study |
| pET28a-His-SUMO-EzrAcyto | pET28a-his-SUMO derivative containing *ezrA*cyto; Kan^R^ | This study |
| pET28a-His-SUMO-EzrA^R514D^cyto | pET28a-his-SUMO derivative containing *ezrA*^R514D^cyto; Kan^R^ | This study |
| pET28a-his-FtsA | pET28a derivative containing his-*ftsA*; Kan^R^ | This study |
| pUT18-*ss*EzrA | P_lac_-T18-*ssezrA* from SC19; Amp^R^ | This study |
| pUT18-*ss*EzrA^R514D^ | P_lac_-T18-*ssezrA*^R514D^ from SC19; Amp^R^ | This study |
| PUT18-*ss*EzrA^ΔQNR^ | P_lac_-T18-*ssezrA*^ΔQNR^ from SC19; Amp^R^ | This study |
| pKT25-*ss*FtsA | P_lac_-T25-*ssftsA* from SC19; Chl^R^ | This study |
| pUT18-*spn*EzrA | P_lac_-T18-*spnezrA* from D39 Δ*cps rpsL*^+^; Amp^R^ | This study |
| pUT18-*spn*EzrA^QND^ | P_lac_-T18-*spnezrA*^QND^ from D39 Δ*cps rpsL*^+^; Amp^R^ | This study |
| pUT18-*spn*EzrA^ΔQNR^ | P_lac_-T18-*spnezrA*^ΔQNR^ from D39 Δ*cps rpsL*^+^; Amp^R^ | This study |
| pKT25-*spn*FtsA | P_lac_-T25-*spnftsA* from D39 Δ*cps rpsL*^+^; Chl^R^ | This study |
| pUT18-*bs*EzrA | P_lac_-T18-*bsezrA* from WB800N; Amp^R^ | This study |
| pUT18-*bs*EzrA^QND^ | P_lac_-T18-*bsezrA*^QND^ from WB800N; Amp^R^ | This study |
| pUT18-*bs*EzrA^ΔQNR^ | P_lac_-T18-*bsezrA*^ΔQNR^ from WB800N; Amp^R^ | This study |
| pKT25-*bs*FtsA | P_lac_-T25-*bsftsA* from WB800N; Chl^R^ | This study |
| pUT18-*sa*EzrA | P_lac_-T18-*saezrA* from RN4220; Amp^R^ | This study |
| pUT18-*sa*EzrA^QND^ | P_lac_-T18-*saezrA*^QND^ from RN4220; Amp^R^ | This study |
| pUT18-*sa*EzrA^ΔQNR^ | P_lac_-T18-*saezrA*^ΔQNR^ from RN4220; Amp^R^ | This study |
| pKT25-*sa*FtsA | P_lac_-T25-*saftsA* from RN4220; Chl^R^ | This study |
| pUT18-*ef*EzrA | P_lac_-T18-*efezrA* from ATCC29212; Amp^R^ | This study |
| pUT18-*ef*EzrA^QND^ | P_lac_-T18-*efezrA*^QND^ from ATCC29212; Amp^R^ | This study |
| pUT18-*ef*EzrA^ΔQNR^ | P_lac_-T18-*ef**ezrA*^ΔQNR^ from ATCC29212; Amp^R^ | This study |
| pKT25-*ef*FtsA | P_lac_-T25-*efftsA* from ATCC29212; Chl^R^ | This study |
| pKT25-FtsZ | P_lac_-T25-*ftsZ* from SC19; Chl^R^ | This study |
| pKT25-SepF | P_lac_-T25-*sepF* from SC19; Chl^R^ | This study |
| pKT25-ZapA | P_lac_-T25-*zapA* from SC19; Chl^R^ | This study |
| pKT25-GpsB | P_lac_-T25-*gpsB* from SC19; Chl^R^ | This study |
| pKT25-MapZ | P_lac_-T25-*mapZ* from SC19; Chl^R^ | This study |
| pKT25-DivIVA | P_lac_-T25-*divIVA* from SC19; Chl^R^ | (7) |
| pKT25-FtsQ | P_lac_-T25-*ftsQ* from SC19; Chl^R^ | This study |
| pKT25-FtsL | P_lac_-T25-*ftsL* from SC19; Chl^R^ | This study |
| pKT25-FtsB | P_lac_-T25-*ftsB* from SC19; Chl^R^ | This study |
| pKT25-StK | P_lac_-T25-*stK* from SC19; Chl^R^ | This study |
| pKT25-RodA | P_lac_-T25-*rodA* from SC19; Chl^R^ | This study |
| pKT25-FtsW | P_lac_-T25-*ftsW* from SC19; Chl^R^ | This study |
| pKT25-PBP1a | P_lac_-T25-*PBP1a* from SC19; Chl^R^ | This study |
| pKT25-PBP2a | P_lac_-T25-*PBP2a* from SC19; Chl^R^ | This study |
| pKT25-PBP1b | P_lac_-T25-*PBP1b* from SC19; Chl^R^ | This study |
| pKT25-PBP2x | P_lac_-T25-*PBP2x* from SC19; Chl^R^ | This study |
| pKT25-PBP2b | P_lac_-T25-*PBP2b* from SC19; Chl^R^ | This study |
| pKT25-MreC | P_lac_-T25-*mreC* from SC19; Chl^R^ | This study |
| pKT25-MreD | P_lac_-T25-*mreD* from SC19; Chl^R^ | This study |
| pKT25-MltG | P_lac_-T25-*mltG* from SC19; Chl^R^ | (7) |
| pKT25-WhiA | P_lac_-T25-*whiA* from SC19; Chl^R^ | This study |
| pKT25-CcrZ | P_lac_-T25-*ccrZ* from SC19; Chl^R^ | This study |
| pKT25-Zip | *Plac*-T25-*leucine zipper* region from yeast GCN4; Chl^R^ | (1) |
| pUT18-Zip | Plac-T18-*leucine zipper* region from yeast GCN4; Amp^R^ | (1) |

**Table S4. Oligonucleotide primers used in this study**

| Primer | Sequence (5’ to 3’) | Template | Amplicon |
| --- | --- | --- | --- |
| For construction of pSET4s-Δ*ezrA* (Δ*ezrA*) | | | |
| Δ*ezrA-*uF | caggtcgactctagaggatccgggaatagtgaagtggtc | SC19 | upstream of *ezrA* |
| Δ*ezrA-*uR | aaagccaaatggctttttatgtaaatctccttagctcata |  |  |
| Δ*ezrA-*dF | tatgagctaaggagatttacataaaaagccatttggcttt | SC19 | downstream of *ezrA* |
| Δ*ezrA-*dR | aaaacgacggccagtgaattcacttgccttactcacatc |  |  |
| For construction of pSET4s-CΔ*ezrA* (CΔ*ezrA*) | | | |
| C*ezrA-*uF | caggtcgactctagaggatcccagctaaatccggtcgcg | SC19 | upstream and 5’ fragment of *ezrA* (L44-CTA replace with CTC) |
| C*ezrA-*uF | cattaactggaaggttaaagagctcttccttacgttc |  |  |
| C*ezrA-*dF | gaacgtaaggaagagctctttaaccttccagttaatg | SC19 | 3’ fragment of *ezrA* (L44-CTA replace with CTC) and downstream of *ezrA* |
| C*ezrA-*dR | aaaacgacggccagtgaattcgagttctttcccgctccc |  |  |
| For construction of pSET4s-*ezrA*^R514D^ (ezrA^R514D^) | | | |
| R514D*-*uF | aggtcgactctagaggatccgttttctgaatttgtgatgct | SC19 | 5’ fragment of *ezrA*^R514D^ |
| R514D*-*uR | tttcatcgaatgaacgataatcgttagagtattgtagcaac |  |  |
| R514D*-*dF | gttgctacaatactctaacgattatcgttcattcgatgaaa | SC19 | *ezrA*^R514D^ and downstream of *ezrA* |
| R514D*-*dR | aaaacgacggccagtgaattcacgtctagcgtcaaaagag |  |  |
| For construction of pSET4s-*ezrA*^ΔQNR^ (*ezrA*^ΔQNR^) | | | |
| R514D*-*uF | aggtcgactctagaggatccgttttctgaatttgtgatgct | SC19 | 5’ fragment of *ezrA*^ΔQNR^ |
| ΔQNR*-*uR | acactttcatcgaatgaacgcaactgctctgtcaaagtc |  |  |
| ΔQNR*-*dF | gactttgacagagcagttgcgttcattcgatgaaagtgt | SC19 | *ezrA*^ΔQNR^ and downstream of *ezrA* |
| R514D*-*dR | aaaacgacggccagtgaattcacgtctagcgtcaaaagag |  |  |
| For construction of pSET4s-*ezrA-mCherry* (EzrA-mCherry); pSET4s-*ezrA*^R514D^-*mCherry* (EzrA^R514D^-mCherry); pSET4s-*ezrA*^ΔQNR^-*mCherry* (EzrA^ΔQNR^-mCherry) | | | |
| Ea-1000-uF | aggtcgactctagaggatcccgggttatcgtaaactgg | SC19/  *ezrA*^R514D^/  *ezrA*^ΔQNR^ | 5’ fragment of *ezrA*/  *ezrA*^R514D^/  *ezrA*^ΔQNR^ |
| Ea-mCh-uR | caccatCTGCAGGAACTCGATGTCTAGTTTgtagcgaatggcttcgcg |  |  |
| Ea-mCh-mF | cgctacAAACTAGACATCGAGTTCCTGCAGatggtgagcaagggcgag | L_2_-mCherry | L_2_-mCherry |
| Ea-mCh-mR | caaatggctttttatctacttgtacagctcgtccat |  |  |
| Ea-mCh-dF | gagctgtacaagtagataaaaagccatttggctt | SC19 | 15 bp 3’ of mCherry, downstream of *ezrA* |
| Δ*ezrA-*dR | aaaacgacggccagtgaattcacttgccttactcacatc |  |  |
| For construction of pSET4s-*ezrA-*3x*flag* (EzrA-3xFLAG); pSET4s-*ezrA*^R514D^*-3xflag* (EzrA^R514D^-3xFLAG); | | | |
| Ea-1000-uF | aggtcgactctagaggatcccgggttatcgtaaactgg | SC19/  *ezrA*^R514D^/ | 5’ fragment of *ezrA*/  *ezrA*^R514D^ |
| Ea-L_0_-3xflag-uR | ggagccagcggaaccgtagcgaatggcttcgc |  |  |
| L_0_-3xflag-F | cgcgaagccattcgctacggttccgctggctccg | synthesized L_0_-*3xflag* | L_0_-*3xflag* |
| L_0_-3xflag-R | aagccaaatggctttttatctaTTTATCATCATCATCTTTATAATCTT |  |  |
| L_0_-3xflag-Ea-dF | TTATAAAGATGATGATGATAAAtagataaaaagccatttggctt | SC19 | 15 bp 3’ of L_0_, downstream of *ezrA* |
| Δ*ezrA-*dR | aaaacgacggccagtgaattcacttgccttactcacatc |  |  |
| For construction of pSET2-P_tetR_-FtsZ-GFP | | | |
| tetR-F | aggtcgactctagaggatccttaagacccactttcacatt | pSSTete2-gfp | P_tetR_ |
| P_tetR_-FtsZ-R | ACTCAAAGGAGAGTAATATAAtggcattttcatttgaagc |  |  |
| P_tetR_-FtsZ-F | ACTCAAAGGAGAGTAATATAAtggcattttcatttgaagc | SC19 | *ftsZ* |
| FtsZ-R | TTACTCATCTGCAGGAACTCGATGTCTAGTTTgcgattacggaagaatgg |  |  |
| FtsZ-L_2_-GFP-F | aatcgcAAACTAGACATCGAGTTCCTGCAGATGAGTAAAGGAGAAGAAC | pSSTete2-gfp | 18 bp 3’ of *ftsZ*, L-gfp |
| GFP-R | aaaacgacggccagtgaattcctaTTTGTATAGTTCATCCATG |  |  |
| For construction of pSET2-P_tetR_-GFP-FtsA | | | |
| tetR-F | aggtcgactctagaggatccttaagacccactttcacatt | pSSTete2-gfp | P_tetR_*-gfp-L_1_* |
| GFP-L_1_-R | aaaagccgtttctagccatTCCGGATCCCTCGAGTT |  |  |
| L_1_-FtsA-F | AACTCGAGGGATCCGGAatggctagaaacggctttt | SC19 | *ftsA* |
| FtsA-R | aaaacgacggccagtgaattcttattcaaacatgctgccaa |  |  |
| For construction of pSET2-P_tetR_-SepF-GFP | | | |
| tetR-F | aggtcgactctagaggatccttaagacccactttcacatt | pSSTete2-gfp | P_tetR_ |
| P_tetR_-SepF-R | taaatgtatcttttaatgccaTTATATTACTCTCCTTTGAGT |  |  |
| P_tetR_-SepF-F | ACTCAAAGGAGAGTAATATAAtggcattaaaagatacattta | SC19 | *sepF* |
| SepF-R | GGAACTCGATGTCTAGTTTccgttttatatcaaattcaaaa |  |  |
| SepF-L_2_-GFP-F | gaatttgatataaaacggAAACTAGACATCGAGTTCC | pSET2-FtsZ-L_2_-GFP | 18 bp 3’ of *sepF*, L_2_-*gfp* |
| GFP-R | aaaacgacggccagtgaattcctaTTTGTATAGTTCATCCATG |  |  |
| For construction of pSET2-P_tetR_-ZapA-GFP | | | |
| tetR-F | aggtcgactctagaggatccttaagacccactttcacatt | pSSTete2-gfp | P_tetR_ |
| P_tetR_-ZapA-R | atatcgatttaagtttgccaTTATATTACTCTCCTTTGAGT |  |  |
| P_tetR_-ZapA-F | ACTCAAAGGAGAGTAATATAAtggcaaacttaaatcgatat | SC19 | *zapA* |
| ZapA-R | GGAACTCGATGTCTAGTTTtaccttgtcctcaagctc |  |  |
| ZapA-L_2_-GFP-F | gagcttgaggacaaggtaAAACTAGACATCGAGTTCC | pSET2-FtsZ-L_2_-GFP | 18 bp 3’ of *zapA*, L_2_-*gfp* |
| GFP-R | aaaacgacggccagtgaattcctaTTTGTATAGTTCATCCATG |  |  |
| For construction of mCherry-*spn*FtsA | | | |
| *spn*FtsA-uF | gcattaccaaggagcaaatag | D39 Δ*cps rpsL*^+^ | upstream of *spnftsA* |
| *spn*FtsA-uR | ctcgcccttgctcaccattacatcgcttcctctct |  |  |
| RBS*_ftsA_*-mCh-L_1_-F | acaaggaagatagagaggaagcgatgtaatggtgagcaagggcgag | *ss*EzrA-mCherry | RBS*_ftsA_*  -mCherry-L_1_ |
| mCh-L_1_-*spn*FtsA-R | TAGCCATTCCGGATCCCTCGAGcttgtacagctcgtccatg |  |  |
| mCh-L_1_-*spn*FtsA-F | TACaagCTCGAGGGATCCGGAatggctagagaaggctttttta | D39 Δ*cps rpsL*^+^ | 3’ fragment of *spnftsA* |
| *spn*FtsA1000-dR | cttcctgagcaagctcaacc |  |  |
| For construction of *spn*EzrA-GFP::Janus | | | |
| *spn*EzrA1000-F | gatttggaagccggttatcg | D39 Δ*cps rpsL*^+^ | 5’ fragment of *spnezrA* |
| *spn*Ea-L_2_-GFP-R | acacgtgaaacgattcgttttAAACTAGACATCGAGTTCCTGCAGATGagc |  |  |
| *spn*Ea-L_2_-GFP-uF | cgttttAAACTAGACATCGAGTTCCTGCAGATGagcaaaggagaagaac | pJWV25 | L_2_-GFP |
| L_2_-GFP-JC-R | AAATCAAACGGATCCTAttatttgtagagctcatccat |  |  |
| JC-F | gagctctacaaataaTAGGATCCGTTTGATTTTTAATG | Pc-[*kan*-*rpsL*+] cassette | Pc-[*kan*-*rpsL*+] |
| JC-R | aatctttttcttttaTTATGCTTTTGGACGTTTAGT |  |  |
| JC-*spn*EzrA-dF | CGTCCAAAAGCATAAtaaaagaaaaagattttattgtgtg | D39 Δ*cps rpsL*^+^ | downstream of *spnezrA* |
| *spn*EzrA-dR | gtgtgtaggaacgacctaact |  |  |
| For construction of *spn*EzrA-GFP | | | |
| *spn*EzrA1000-F | gatttggaagccggttatcg | *spn*EzrA-GFP::Janus | *spnezrA*-*gfp* |
| L_2_-GFP-R | aatctttttcttttattatttgtagagctcatccat |  |  |
| GFP-*spn*EzrA-dF | gagctctacaaataataaaagaaaaagattttattgtgtg | D39 Δ*cps rpsL*^+^ | downstream of *spnezrA* |
| *spn*EzrA-dR | gtgtgtaggaacgacctaact |  |  |
| For construction of *spn*EzrA^QND^-GFP::Janus | | | |
| *spn*EzrA1000-F | gatttggaagccggttatcg | D39 Δ*cps rpsL*^+^ | 5’ fragment with *spnezrA*^QND^ |
| *spn*QND-R | catcaaatgagcgataATCgttagaatattgcaagagttg |  |  |
| *spn*QND-F | cttgcaatattctaacGATtatcgctcatttgatgaacg | *spn*EzrA-GFP::Janus | *spnezrA*^QND^- Pc-[*kan*-*rpsL*+] and downstream |
| *spn*EzrA-dR | gtgtgtaggaacgacctaact |  |  |
| For construction of *spn*EzrA^QND^-GFP | | | |
| *spn*EzrA1000-F | gatttggaagccggttatcg | *spn*EzrA^QND^-GFP::Janus | *spnezrA*^QND^-L_2_-*gfp* |
| L_2_-GFP-R | aatctttttcttttattatttgtagagctcatccat |  |  |
| GFP-*spn*EzrA-dF | gagctctacaaataataaaagaaaaagattttattgtgtg | D39 Δ*cps rpsL*^+^ | downstream of *spnezrA* |
| *spn*EzrA-dR | gtgtgtaggaacgacctaact |  |  |
| For construction of P_Zn_-mCherry-*spn*FtsA | | | |
| gatC-F | cgccccaagttcatcaccAA | pJWV25 | *bgaA*’::*tet*-PZn*-*RBS*_ftsA_* |
| PZn*-*RBS*_ftsA_-*R | attacatcgcttcctctctatcttccttgttataatagatttatgaacacctt |  |  |
| RBS*_ftsA_*-mCh-L_1_-F | acaaggaagatagagaggaagcgatgtaatggtgagcaagggcgag | ssEzrA-mCherry | RBS*_ftsA_*  -mCherry-L_1_ |
| mCh-L_1_-*spn*FtsA-R | TAGCCATTCCGGATCCCTCGAGcttgtacagctcgtccatg |  |  |
| mCh-L_1_-*spn*FtsA-F | TACaagCTCGAGGGATCCGGAatggctagagaaggctttttta | D39 Δ*cps rpsL*^+^ | *spn*FtsA |
| *spn*FtsA-R | tatgagaaagtaagttcttattcgtcaaacatgcttcc |  |  |
| bgaA-F | agcatgtttgacgaataagaacttactttctcataaaccag | D39 Δ*cps rpsL*^+^ | 3’ fragment containing *bgaA’* |
| bgaA-R | gctttcttgaggcaattcactt |  |  |
| For construction of pET28a-His-SUMO-EzrAcyto | | | |
| SUMO-EzrAcyto-F | agagaacagattggtGGATCCcgtaaacgaaatgacaacc | SC19 | *ssezrA*cyto |
| 28a-EzrA-R | CGGAGCTCGAATTCGGATCCctagtagcgaatggcttc |  |  |
| For construction of pET28a-His-SUMO-EzrA^R514D^cyto | | | |
| SUMO-EzrAcyto-F | agagaacagattggtGGATCCcgtaaacgaaatgacaacc | SC19 | *ezrA*^R514D^cyto |
| 28a-EzrA-R | CGGAGCTCGAATTCGGATCCctagtagcgaatggcttc |  |  |
| For construction of pET28a-his-FtsA | | | |
| 28a-FtsA-F | GGCCTGGTGCCGCGCGGCAGCatggctagaaacggctttt | SC19 | *ftsA* |
| 28a-FtsA-R | ACCAGTCATGCTAGCCATATGttattcaaacatgctgccaa |  |  |
| For construction of pUT18-*ss*EzrA | | | |
| T18-*ss*Ea-F | gtaccgggccccccctcgaggAtgcctacaggaacaatcat | SC19 | *ssezrA* |
| T18-*ss*Ea-R | aagcttatcgataccgtcgacctgtagcgaatggcttcgcg |  |  |
| For construction of pUT18-*ss*EzrA^R514D^ | | | |
| T18-*ss*Ea-F | gtaccgggccccccctcgaggAtgcctacaggaacaatcat | *ssezrA*^R514D^ | *ssezrA*^R514D^ |
| T18-*ss*Ea-R | aagcttatcgataccgtcgacctgtagcgaatggcttcgcg |  |  |
| For construction of pUT18-*ss*EzrA^ΔQNR^ | | | |
| T18-*ss*Ea-F | gtaccgggccccccctcgaggAtgcctacaggaacaatcat | *ssezrA*^ΔQNR^ | *ssezrA*^ΔQNR^ |
| T18-*ss*Ea-R | aagcttatcgataccgtcgacctgtagcgaatggcttcgcg |  |  |
| For construction of pKT25-*ss*FtsA | | | |
| T25-*ss*FtsA-F | GGGTCGACTCTAGAGGATCCCatggctagaaacggctttt | SC19 | *ssftsA* |
| T25-*ss*FtsA-R | TACTTAGGTACCCGGGGATCCttattcaaacatgctgccaa |  |  |
| For construction of pUT18-*spn*EzrA | | | |
| T18-*spn*EzrA-F | TACCGGGCCCCCCCTCGAGGatgtctaatggacaactaattt | D39 Δ*cps rpsL*^+^ | *spnezrA* |
| T18-*spn*EzrA-R | CAAGCTTATCGATACCGTCGAaaaacgaatcgtttcacgtg |  |  |
| For construction of pUT18-*spn*EzrA^QND^ | | | |
| T18-*spn*EzrA-F | TACCGGGCCCCCCCTCGAGGatgtctaatggacaactaattt | *spn*EzrA^QND^-GFP | *spnezrA*^QND^ |
| T18-*spn*EzrA-R | CAAGCTTATCGATACCGTCGAaaaacgaatcgtttcacgtg |  |  |
| For construction of pUT18-*spn*EzrA^ΔQNR^ | | | |
| T18-*spn*EzrA-F | TACCGGGCCCCCCCTCGAGGatgtctaatggacaactaattt | D39 Δ*cps rpsL*^+^ | 5’ fragment of *spnezrA*^ΔQNR^ |
| *spn*ΔQNR-R | aatgcgttcatcaaatgagagttgctctgtcaaagttg |  |  |
| *spn*ΔQNR-F | ctttgacagagcaactctcatttgatgaacgcattcaa | D39 Δ*cps rpsL*^+^ | 3’ fragment of *spnezrA*^ΔQNR^ |
| T18-*spn*EzrA-R | CAAGCTTATCGATACCGTCGAaaaacgaatcgtttcacgtg |  |  |
| For construction of pKT25-*spn*FtsA | | | |
| T25-*spn*FtsA-F | CTGCAGGGTCGACTCTAGAGatggctagagaaggcttttt | D39 Δ*cps rpsL*^+^ | *spnftsA* |
| T25-*spn*FtsA-R | CTTACTTAGGTACCCGGGGAttattcgtcaaacatgcttc |  |  |
| For construction of pUT18-*bs*EzrA | | | |
| T18-*bs*EzrA-F | TACCGGGCCCCCCCTCGAGGatggagtttgtcattggattat | WB800N | *bsezrA* |
| T18-*bs*EzrA-R | CAAGCTTATCGATACCGTCGAagcggatatgtcagcttt |  |  |
| For construction of pUT18-*bs*EzrA^QND^ | | | |
| T18-*bs*EzrA-F | TACCGGGCCCCCCCTCGAGGatggagtttgtcattggattat | WB800N | 5’ fragment of *bsezrA*^QND^ |
| *bs*QND-R | gattctggcttctgaaATCgtttccgaactgaatgatttt |  |  |
| *bs*QND-F | cattcagttcggaaacGATttcagaagccagaatcatatt | WB800N | 3’ fragment of *bsezrA*^QND^ |
| T18-bsEzrA-R | CAAGCTTATCGATACCGTCGAagcggatatgtcagcttt |  |  |
| For construction of pUT18-*bs*EzrA^ΔQNR^ | | | |
| T18-*bs*EzrA-F | TACCGGGCCCCCCCTCGAGGatggagtttgtcattggattat | WB800N | 5’ fragment of *bsezrA*^ΔQNR^ |
| *bs*ΔQNR-R | aaaatatgattctggctgattttttcaatgaggatgac |  |  |
| *bs*ΔQNR-F | cctcattgaaaaaatcagccagaatcatattttatctg | WB800N | 3’ fragment of *bsezrA*^ΔQNR^ |
| T18-*bs*EzrA-R | CAAGCTTATCGATACCGTCGAagcggatatgtcagcttt |  |  |
| For construction of pKT25-*bs*FtsA | | | |
| T25-*bs*FtsA-F | CTGCAGGGTCGACTCTAGAGatgaacaacaatgaactttacg | WB800N | *bsftsA* |
| T25-*bs*FtsA-R | CTTACTTAGGTACCCGGGGActattcccaaaacatgcttaat |  |  |
| For construction of pUT18-*sa*EzrA | | | |
| T18-*sa*EzrA-F | TACCGGGCCCCCCCTCGAGGatggtgttatatatcattttgg | RN4220 | *saezrA* |
| T18-*sa*EzrA-R | CAAGCTTATCGATACCGTCGAttgcttaataacttcttcttca |  |  |
| For construction of pUT18-*sa*EzrA^QND^ | | | |
| T18-*sa*EzrA-F | TACCGGGCCCCCCCTCGAGGatggtgttatatatcattttgg | RN4220 | 5’ fragment of *saezrA*^QND^ |
| *sa*QND-R | tatagtccttacgataATCatttccatattgaattaatttctc |  |  |
| *sa*QND-F | aattcaatatggaaatGATtatcgtaaggactatagcaat | RN4220 | 3’ fragment of *saezrA*^QND^ |
| T18-*sa*EzrA-R | CAAGCTTATCGATACCGTCGAttgcttaataacttcttcttca |  |  |
| For construction of pUT18-*sa*EzrA^ΔQNR^ | | | |
| T18-*sa*EzrA-F | TACCGGGCCCCCCCTCGAGGatggtgttatatatcattttgg | RN4220 | 5’ fragment of *saezrA*^ΔQNR^ |
| *sa*ΔQNR-R | aacattgctatagtcctttaatttctctgcataaacagc |  |  |
| *sa*ΔQNR-F | tttatgcagagaaattaaaggactatagcaatgttgat | RN4220 | 3’ fragment of *saezrA*^ΔQNR^ |
| T18-*sa*EzrA-R | CAAGCTTATCGATACCGTCGAttgcttaataacttcttcttca |  |  |
| For construction of pKT25-*sa*FtsA | | | |
| T25-*sa*FtsA-F | CTGCAGGGTCGACTCTAGAGatggaagaacattactacgtaa | RN4220 | *saftsA* |
| T25*-sa*FtsA-R | CTTACTTAGGTACCCGGGGAtcattcaaatagagatttcatta |  |  |
| For construction of pUT18-*ef*EzrA | | | |
| T18-*ef*EzrA-F | TACCGGGCCCCCCCTCGAGGttggattatcattttagtactagt | ATCC29212 | *efezrA* |
| T18-*ef*EzrA-R | AAGCTTATCGATACCGTCGAaattgctgttaaattagggttat |  |  |
| For construction of pUT18-*ef*EzrA^QND^ | | | |
| T18-*ef*EzrA-F | TACCGGGCCCCCCCTCGAGGttggattatcattttagtactagt | ATCC29212 | 5’ fragment of *efezrA*^QND^ |
| *ef*QND-R | cgtgagtatgacgataGTCgtttgcatattgcatcatttg |  |  |
| *ef*QND-F | gatgcaatatgcaaacGACtatcgtcatactcacgag | ATCC29212 | 3’ fragment of *efezrA*^QND^ |
| T18-*ef*EzrA-R | AAGCTTATCGATACCGTCGAaattgctgttaaattagggttat |  |  |
| For construction of pUT18-*ef*EzrA^ΔQNR^ | | | |
| T18-*ef*EzrA-F | TACCGGGCCCCCCCTCGAGGttggattatcattttagtactagt | ATCC29212 | 5’ fragment of *efezrA*^ΔQNR^ |
| *ef*ΔQNR-R | atgttctcgtgagtatgcatttgttctgtcagagct |  |  |
| *ef*ΔQNR-F | ctctgacagaacaaatgcatactcacgagaacatcc | ATCC29212 | 3’ fragment of *efezrA*^ΔQNR^ |
| T18-*ef*EzrA-R | AAGCTTATCGATACCGTCGAaattgctgttaaattagggttat |  |  |
| For construction of pUT18-*ef*FtsA | | | |
| T18-*ef*FtsA-F | CTGCAGGGTCGACTCTAGAGatggcaaaaacaggaatgtat | ATCC29212 | *efftsA* |
| T18-*ef*FtsA-R | CTTACTTAGGTACCCGGGGAttagtcgaaaatgttcgagaa |  |  |
| For construction of pKT25-FtsZ | | | |
| T25-FtsZ-F | agggtcgactctagaggatccCAtggcattttcatttgaagc | SC19 | *ftsZ* |
| T25-FtsZ-R | tacttaggtacccggggatccttagcgattacggaagaatgg |  |  |
| For construction of pKT25-SepF | | | |
| T25-SepF-F | agggtcgactctagaggatccCAtggcattaaaagatacattta | SC19 | *sepF* |
| T25-SepF-R | tacttaggtacccggggatccttaccgttttatatcaaattca |  |  |
| For construction of pKT25-ZapA | | | |
| T25-ZapA-F | agggtcgactctagaggatcccAtggcaaacttaaatcgatat | SC19 | *zapA* |
| T25-ZapA-R | tacttaggtacccggggatcctcataccttgtcctcaag |  |  |
| For construction of pKT25-GpsB | | | |
| T25-GpsB-F | agggtcgactctagaggatccCatggcaagtattaaatttacg | SC19 | *gpsB* |
| T25-GpsB-R | tacttaggtacccggggatccctattcttgatcctgaacg |  |  |
| For construction of pKT25-MapZ | | | |
| T25-MapZ-F | agggtcgactctagaggatccCgtgggagaaaaaaatagtca | SC19 | *mapZ* |
| T25-MapZ-R | tacttaggtacccggggatccttaataatccaaggcatcaga |  |  |
| For construction of pKT25-FtsQ | | | |
| T25-FtsQ-F | agggtcgactctagaggatccCatgacggaaaaagattcaaat | SC19 | *ftsQ* |
| T25-FtsQ-R | tacttaggtacccggggatcctcagcttgcatatctataaat |  |  |
| For construction of pKT25-FtsL | | | |
| T25-FtsL-F | agggtcgactctagaggatcccAtgttgcaagaaaaacgtag | SC19 | *ftsL* |
| T25-FtsL-R | tacttaggtacccggggatccttattcggctactccaatatt |  |  |
| For construction of pKT25-FtsB | | | |
| T25-FtsB-F | agggtcgactctagaggatcccatgaagaaatctaaaattctac | SC19 | *ftsB* |
| T25-FtsB-R | tacttaggtacccggggatcctcattttggtaaccctgga |  |  |
| For construction of pKT25-StK | | | |
| T25-StK-F | agggtcgactctagaggatccCatgattcaaatcggtaagatc | SC19 | *stK* |
| T25-StK-R | tacttaggtacccggggatccttattgtccgctacctgtt |  |  |
| For construction of pKT25-RodA | | | |
| T25-RodA-F | agggtcgactctagaggatccCAtgaggaaattgatgaaaaata | SC19 | *rodA* |
| T25-RodA-R | tacttaggtacccggggatccctaacgttttatcgtaattttt |  |  |
| For construction of pKT25-FtsW | | | |
| T25-FtsW-F | agggtcgactctagaggatccCAtgaagattgacaaacgcca | SC19 | *ftsW* |
| T25-FtsW-R | tacttaggtacccggggatccctaaattagcgtacgctcta |  |  |
| For construction of pKT25-PBP1a | | | |
| T25-PBP1a-F | agggtcgactctagaggatccCttgaaaactacaacaattaaaaa | SC19 | *PBP1a* |
| T25-PBP1a-R | tacttaggtacccggggatccttattgaccatctgtctgtc |  |  |
| For construction of pKT25-PBP2a | | | |
| T25-PBP2a-F | agggtcgactctagaggatccCatggacgatttgcaacatc | SC19 | *PBP2a* |
| T25-PBP2a-R | tacttaggtacccggggatccttatggatttgacaactctc |  |  |
| For construction of pKT25-PBP1b | | | |
| T25-PBP1b-F | agggtcgactctagaggatccCatggcgactaaatcagataa | SC19 | *PBP1b* |
| T25-PBP1b-R | tacttaggtacccggggatcctcaaggtgaatcattattcg |  |  |
| For construction of pKT25-PBP2x | | | |
| T25-PBP2x-F | agggtcgactctagaggatccCatgcccagaagaaataataaa | SC19 | *PBP2x* |
| T25-PBP2x-R | tacttaggtacccggggatcctcagtctcctaaggttattg |  |  |
| For construction of pKT25-PBP2b | | | |
| T25-PBP2b-F | agggtcgactctagaggatccCgtgaaaaaaagaaagaacaag | SC19 | *PBP2b* |
| T25-PBP2b-R | tacttaggtacccggggatccttaattcattgggtgaagtg |  |  |
| For construction of pKT25-MreC | | | |
| T25-MreC-F | agggtcgactctagaggatccCatgaataaattttcaaaattagta | SC19 | *mreC* |
| T25-MreC-R | tacttaggtacccggggatccttattgcgcatttgtttgac |  |  |
| For construction of pKT25-MreD | | | |
| T25-MreD-F | agggtcgactctagaggatcccAtgcgcaataaaatgatagaa | SC19 | *mreD* |
| T25-MreD-R | tacttaggtacccggggatccctatgtcttgtttgttattc |  |  |
| For construction of pKT25-WhiA | | | |
| T25-WhiA-F | agggtcgactctagaggatccCAtgagttttacagtacaagta | SC19 | *whiA* |
| T25-WhiA-R | tacttaggtacccggggatccCTAgtcagtcaattcatccgc |  |  |
| For construction of pKT25-CcrZ | | | |
| T25-CcrZ-F | agggtcgactctagaggatccCAtgcagtttgataccagtg | SC19 | *ccrZ* |
| T25-CcrZ-R | tacttaggtacccggggatcctcatacttccttatagaaact |  |  |

^a^ Linkers and tags are annotated as described below. L_0_ (GSAGSAAGSG) is made to the C-termina of 3xFLAG-tagged proteins, L_1_ (LEGSG) is used for N-termina of fluorescent fusion-tagged protein, like GFP-L_1_-*ss*FtsA (8), L_2_ (KLDIEFLQ) is made to C-termina of fluorescent fusion-tagged protein, such as *spn*EzrA-L_2_-GFP and *ss*FtsZ-L_2_-GFP (8), and FLAG sequence is DYKDDDDK (9).

^b^ Antibiotic resistance markers: Spc^R^, spectinomycin; Kan^R^, kanamycin; Str^R^, streptomycin; Chl^R^, chloramphenicol; Tet^R^, tetracycline.

^c^ Strain of Janus::*spn*FtsA was constructed according to the study from Andrea Mura (4).

^d^ Because *spnezrA* is essential for the basic growth of pneumococcal cells, therefore, the fused fragment (*spn*EzrA^QND^-GFP-Janus and downstream of *spnezrA*) was introduced into the strain of Δ*cps rpsL* by natural transformation. Then *spn*EzrA^QND^-gfp::Janus strain was confirmed by DNA sequencing.

SUPPLEMENTARY REFERENCES

1. Karimova G, Pidoux J, Ullmann A, Ladant D. 1998. A bacterial two-hybrid system based on a reconstituted signal transduction pathway. Proc Natl Acad Sci U S A 95:5752-6.

2. Li W, Liu L, Chen H, Zhou R. 2009. Identification of Streptococcus suis genes preferentially expressed under iron starvation by selective capture of transcribed sequences. FEMS Microbiol Lett 292:123-33.

3. Lanie JA, Ng WL, Kazmierczak KM, Andrzejewski TM, Davidsen TM, Wayne KJ, Tettelin H, Glass JI, Winkler ME. 2007. Genome sequence of Avery's virulent serotype 2 strain D39 of Streptococcus pneumoniae and comparison with that of unencapsulated laboratory strain R6. J Bacteriol 189:38-51.

4. Mura A, Fadda D, Perez AJ, Danforth ML, Musu D, Rico AI, Krupka M, Denapaite D, Tsui HT, Winkler ME, Branny P, Vicente M, Margolin W, Massidda O. 2017. Roles of the Essential Protein FtsA in Cell Growth and Division in Streptococcus pneumoniae. J Bacteriol 199.

5. Takamatsu D, Osaki M, Sekizaki T. 2001. Thermosensitive suicide vectors for gene replacement in Streptococcus suis. Plasmid 46:140-8.

6. Zhang L, Zou W, Ni M, Hu Q, Zhao L, Liao X, Huang Q, Zhou R. 2022. Development and Application of Two Inducible Expression Systems for Streptococcus suis. Microbiol Spectr 10:e0036322.

7. Jiang Q, Li B, Zhang L, Li T, Hu Q, Li H, Zou W, Hu Z, Huang Q, Zhou R. 2023. DivIVA Interacts with the Cell Wall Hydrolase MltG To Regulate Peptidoglycan Synthesis in Streptococcus suis. Microbiol Spectr 11:e0475022.

8. Fleurie A, Manuse S, Zhao C, Campo N, Cluzel C, Lavergne JP, Freton C, Combet C, Guiral S, Soufi B, Macek B, Kuru E, VanNieuwenhze MS, Brun YV, Di Guilmi AM, Claverys JP, Galinier A, Grangeasse C. 2014. Interplay of the serine/threonine-kinase StkP and the paralogs DivIVA and GpsB in pneumococcal cell elongation and division. PLoS Genet 10:e1004275.

9. Wayne KJ, Sham LT, Tsui HC, Gutu AD, Barendt SM, Keen SK, Winkler ME. 2010. Localization and cellular amounts of the WalRKJ (VicRKX) two-component regulatory system proteins in serotype 2 Streptococcus pneumoniae. J Bacteriol 192:4388-94.
